# Histone chaperone exploits intrinsic disorder to switch acetylation specificity

**DOI:** 10.1038/s41467-019-11410-7

**Published:** 2019-08-06

**Authors:** Nataliya Danilenko, Lukas Lercher, John Kirkpatrick, Frank Gabel, Luca Codutti, Teresa Carlomagno

**Affiliations:** 1 Leibniz University Hannover, Centre for Biomolecular Drug Research, Schneiderberg 38, D-30167 Hannover, Germany; 2 Helmholtz Centre for Infection Research, Group of Structural Chemistry, Inhoffenstrasse 7, D-38124 Braunschweig, Germany; 3 University Grenoble Alpes, CEA, CNRS IBS, 71 avenue des Martyrs, F-38044 Grenoble, France; 4 Institut Laue-Langevin, 71 avenue des Martyrs, F-38042 Grenoble, France

**Keywords:** Post-translational modifications, Structural biology

## Abstract

Histones, the principal protein components of chromatin, contain long disordered sequences, which are extensively post-translationally modified. Although histone chaperones are known to control both the activity and specificity of histone-modifying enzymes, the mechanisms promoting modification of highly disordered substrates, such as lysine-acetylation within the N-terminal tail of histone H3, are not understood. Here, to understand how histone chaperones Asf1 and Vps75 together promote H3 K9-acetylation, we establish the solution structural model of the acetyltransferase Rtt109 in complex with Asf1 and Vps75 and the histone dimer H3:H4. We show that Vps75 promotes K9-acetylation by engaging the H3 N-terminal tail in fuzzy electrostatic interactions with its disordered C-terminal domain, thereby confining the H3 tail to a wide central cavity faced by the Rtt109 active site. These fuzzy interactions between disordered domains achieve localization of lysine residues in the H3 tail to the catalytic site with minimal loss of entropy, and may represent a common mechanism of enzymatic reactions involving highly disordered substrates.

## Abstract (web summary)

Histone chaperones have been shown to control the activity and specificity of histone-modifying enzymes. Here the authors establish a structural model of the acetyltransferase Rtt109 in complex with Asf1 and Vps75 and the histone dimer H3:H4, finding that Vps75 promotes K9-acetylation by engaging the H3 N-terminal tail in fuzzy electrostatic interactions with its disordered C-terminal domain.

## Introduction

Eukaryotic DNA is packaged into chromatin, a large molecular assembly consisting principally of nucleosomes. These comprise ~145 base-pairs of DNA wrapped around a protein octamer formed by two copies of each of the histones H2A, H2B, H3, and H4^1^. Histones are subject to extensive post-translational modifications (PTMs), which are involved in the regulation of cellular processes, such as transcription and DNA repair^2^. Acetylation of lysine side-chains is a common PTM that can either directly modulate chromatin structure or provide a binding site for histone-modifying enzymes and remodeling machineries^3,4^. Acetylation of K56 on histone H3 (H3-K56ac), located just before the H3 core, is required for genomic stability^5^ and nucleosome assembly^6^. Additionally, the N-terminal tail of histone H3 can be acetylated at multiple sites, including K9, K14, K23, and K27. K9-acetylation (H3-K9ac) is associated with actively transcribed regions and has been proposed to promote histone eviction from the nucleosome^7^. In both yeast and humans, H3-K9ac is enriched at the promoters and the 5′-ends of genes^8^.

The incorporation of PTMs is highly regulated^9^. In yeast, the acetyltransferase Rtt109 (regulator of Ty1 transposition 109) acetylates newly synthesized histone H3 on both K56 and K9^10–12^. Despite the lack of sequence homology, the catalytic domain of the human ortholog of Rtt109, the acetyltransferase p300/CBP^13^, adopts a similar three-dimensional fold^14–16^. Rtt109 shows very little activity on isolated histones, but efficiently acetylates H3:H4 dimers associated with the histone chaperones Asf1 (anti-silencing function protein 1) and Vps75 (vacuolar protein sorting-associated protein 75)^10,11,17,18^. Indeed, both the activity and specificity of Rtt109 in vivo and in vitro is dependent on the identity of the associated chaperones^12,19,20^.

The histone chaperone Asf1, conserved from yeast to humans, binds the H3:H4 dimer, thus preventing formation of the (H3:H4)_2_ tetramer^21,22^. In complex with H3:H4, Asf1 is required for both H3-K56ac and H3-K9ac^12,19,23^. The crystal structure of the Asf1–H3:H4–Rtt109 complex from *Aspergillus fumigatus* (*Af*)^24^ shows that Asf1 promotes binding of Rtt109 to the H3:H4 core by stabilizing the conformation of the H4 C-terminal region. In the structure, H3-K56 is found to extend into the Rtt109 catalytic site, but there is no well-defined electron-density for the H3 N-terminal tail, suggesting that it remains disordered and does not bind to Rtt109^24^.

The histone chaperone Vps75 belongs to the nucleosome assembly protein (NAP-1) family, which can bind to the four core histones (H2A, H2B, H3, and H4)^25^. Like Asf1, Vps75 stimulates acetylation of H3 by Rtt109^10^; however, unlike Asf1, it is critical for H3-K9ac but not for H3-K56ac, and can also facilitate acetylation of K23 and K27^12,17,26^.

A marked difference between these acetylation sites is that K9, K23, and K27 are in the long, unstructured N-terminal tail, whereas K56 is located immediately before the start of the structured H3 core domain. In the Asf1–H3:H4–Rtt109 complex, Rtt109 binds the H3:H4 core, thereby bringing H3-K56 in proximity to the Rtt109 active center. In contrast, the long, disordered linkers that separate H3-K9, K23 or K27 from the first residue of the H3 core (L60) sample a great many conformations, in most of which the sites to be acetylated are far from the Rtt109 catalytic pocket. Numerous protein–protein interactions between disordered sequences and folded domains have been described, in which the disordered sequence is either recognized in its unfolded state or folds upon binding^27,28^. Upon addition of Vps75 to the Asf1–H3:H4–Rtt109 complex, interactions between the disordered H3 N-terminal domain and the folded core domains of the chaperones could theoretically promote the recruitment of the acetylation sites in the H3 tail to Rtt109. However, this mechanism would require a large surface of Vps75 to be available for interaction with the long H3 N-terminal domain. Partial folding of this domain would moderate the demand on the size of the interaction surface, but would imply a greater loss of entropy. Ultimately, the mechanisms by which Vps75 and Asf1 act in concert to promote acetylation of lysine side-chains within the H3 tail remain elusive.

Here, to discover how Asf1 and Vps75 work together to support K9-acetylation, we establish a structural model for the 160-kDa Asf1–H3:H4–Rtt109–Vps75_2_ complex in solution to a precision of 2.0 Å, using a combination of nuclear magnetic resonance (NMR)-derived distance restraints, small-angle neutron scattering (SANS) and molecular dynamics (MD) simulations. Our study demonstrates that Vps75 promotes acetylation of K9 via a two-fold mechanism, which differs from a classical interaction between folded and unfolded protein domains. First, the Vps75 dimer acts as a binding platform for both the Asf1–H3:H4 substrate and the Rtt109 enzyme, building a doughnut-like structure with a 25-Å-wide central cavity faced by the Rtt109 catalytic site. Second, the unstructured C-terminal tail of Vps75 (C-terminal acidic domain, CTAD) recruits the similarly unstructured H3 N-terminal tail, containing K9, to this cavity via fuzzy electrostatic interactions. Both the Vps75 CTAD and the H3 N-terminal domain remain disordered, allowing confinement of the H3 tail in proximity to the enzyme active site with minimal entropic penalty. The plasticity of the electrostatic contacts between the Vps75 CTAD and the H3 tail, which derives from the disordered nature of both interacting partners, allows any one of K9, K23 or K27 to enter the Rtt109 catalytic pocket, thereby ensuring the availability of all acetylation sites within the H3 tail. We propose that the mechanism discovered here may be a general feature of enzymatic reactions involving long, unstructured protein substrates.

## Results

### Asf1–H3:H4–Rtt109–Vps75_2_ has a doughnut-like shape

**Fig. 1:**
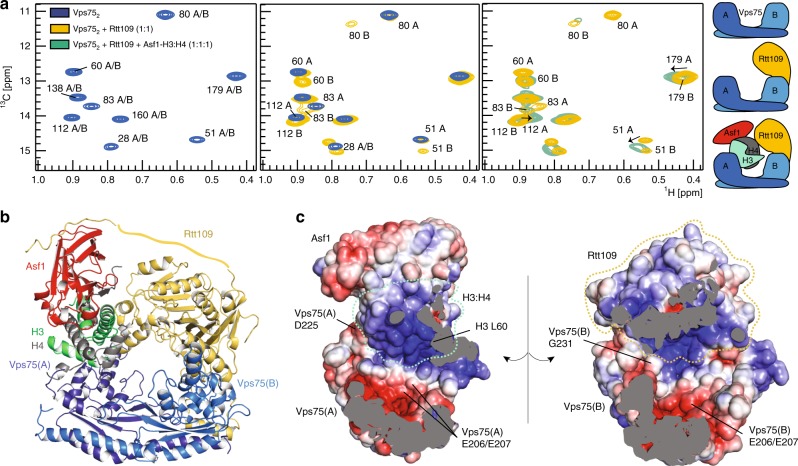
Vps75_2_ forms a doughnut-shaped complex with Asf1, H3:H4 and Rtt109. **a** Overlay of ^1^H-^13^C HMQC spectra of ILV methyl-labeled Vps75_2_ in isolation, with Rtt109 and in the Asf1–H3:H4–Rtt109–Vps75_2_ complex. In Vps75_2_, Vps75(A) and Vps75(B) have identical chemical shifts (left). In complex with Rtt109 (middle), only Vps75(B) displays CSPs. Upon further addition of Asf1–H3:H4 (right), the Vps75(B) peaks do not shift further, while Vps75(A) displays noticeable CSPs. Thus, Vps75(B) recruits Rtt109, while Vps75(A) binds Asf1–H3:H4. Spectra were recorded on samples of 60 μM Vps75_2_ in 50 mM sodium citrate pH 6.5, 150 mM NaCl, 5 mM BME at 850 MHz and 298 K. **b** The Asf1–H3:H4–Rtt109–Vps75_2_ complex adopts a doughnut-like shape with a central cavity of ~25 Å width. The Rtt109 C-terminal tail (Rtt109^419–433^) is shown bound to Asf1, as described in Lercher et al.^34^ (PDB entry 6f0y). A flat ribbon indicates the portion of the Rtt109 C-terminal tail for which no structural information is available. The disordered Vps75 and H3 tails are not shown. **c** Electrostatic surface representation of the inner part of the complex. D225 and G231 are the last structured amino acids of Vps75_2_; L60 is the first amino acid of the H3 core; the Vps75-^206^EE^207^ dyad is at the center of an acidic patch

We assembled a stable complex comprised of *Saccharomyces cerevisiae* (*Sc*) Asf1, *Xenopus laevis* H3:H4, *Sc*Rtt109, and *Sc*Vps75 from individual recombinant proteins. The full complex was isolated by size-exclusion chromatography (Supplementary Fig. 1, Methods). *Xenopus* histones are commonly used in structural studies instead of yeast histones because of their high expression levels and near-perfect sequence identity with yeast histones (89% for H3 and 92% for H4; Supplementary Fig. 2). Published crystal structures^29,30^ have shown that Rtt109 can bind Vps75 in either a 1:2 or a 2:2 stoichiometric ratio. In our hands, both multiple-angle light-scattering data (Supplementary Fig. 1a, b) and titration of the Vps75_2_ homodimer with Rtt109 monitored by NMR with 2D ^1^H-^13^C-methyl spectra identified a stable Rtt109–Vps75_2_ complex, confirming the 1:2 stoichiometry. A small proportion of the Rtt109_2_–Vps75_2_ complex could be observed in NMR spectra only when mixing equimolar amounts of Rtt109 and Vps75 at concentrations above 20 μM. Addition of Asf1–H3:H4 to the mixture of Rtt109 and Vps75 yielded a homogeneous Asf1–H3:H4–Rtt109–Vps75_2_ complex, irrespective of the initial ratio of Rtt109 to Vps75. This indicates that the Rtt109_2_–Vps75_2_ complex is abolished by addition of Asf1–H3:H4. In the Asf1–H3:H4–Rtt109–Vps75_2_ complex, one subunit of the Vps75 homodimer binds to the substrate Asf1–H3:H4 (Vps75(A)), while the second subunit (Vps75(B)) recruits the catalytic module Rtt109 (Fig. 1, Supplementary Fig. 1).

We determined the structural model of the 160-kDa hexameric Asf1–H3:H4–Rtt109–Vps75_2_ complex in solution using the M3 structure-calculation protocol^31,32^. M3 is a data-driven docking routine that calculates structural models of complexes from semi-flexible sub-complex structures. As structural data, we used NMR-derived distance restraints and SANS curves recorded with contrast-matching for multiple combinations of ^1^H- and ^2^H-labeled Asf1, H3:H4, Rtt109, and Vps75.

NMR spectroscopy of high-molecular-weight systems focuses on the signals of selectively ^1^H,^13^C-labeled methyl groups of methyl-bearing residues in the context of perdeuterated proteins. These methyl NMR signals remain detectable at molecular-weights above 100 kDa due to their favorable relaxation properties and the high intensity of the signal generated by the three equivalent hydrogens. Here, distance restraints were obtained from paramagnetic relaxation enhancement (PRE) experiments detected on isoleucine, leucine, and valine (ILV) methyl groups. Cysteine residues were engineered at specific positions on H3:H4 and coupled to a paramagnetic tag (Supplementary Fig. 3); each sample contained a single tag. The relaxation enhancements induced by the paramagnetic center on the methyl groups of ILV-labeled Vps75 or I-labeled Rtt109 residues were quantified and converted into distances^33^ (Methods).

For the SANS experiments we assembled the full complex using truncated versions of H3 and Vps75 (Asf1–H3^35–135^:H4–Rtt109–Vps75_2_^1–225^; Supplementary Fig. 4). The SANS curves of this complex reported on the relative position and orientation of folded subunits without being perturbed by the signal arising from the long disordered Vps75 CTAD (Vps75^226–264^) and H3 N-terminal tail (H3^1–34^). The Asf1–H3^35–135^:H4–Rtt109–Vps75_2_^1–225^ complex displayed an identical Rtt109–Asf1 SANS curve and Vps75 NMR chemical shift perturbations (CSPs) to the complex assembled with full-length proteins (Supplementary Fig. 1c–e), indicating that the truncation of the Vps75 and H3 tails does not disturb the complex geometry.

As building blocks for M3, we used the crystal structure of the Asf1–H3:H4 sub-complex (PDB entry 2hue)^21^ and MD-generated structures of the Rtt109–Vps75_2_ sub-complex. To verify that the structure of the Asf1–H3:H4 sub-complex is preserved upon formation of the Asf1–H3:H4–Rtt109–Vps75_2_ complex, we employed a combination of NMR and SANS data. The chemical shifts of Asf1 signals arising from residues at the interface with H3:H4 in Asf1–H3:H4 were identical in Asf1–H3:H4–Rtt109–Vps75_2_, indicating that the interface between Asf1 and the H3:H4 dimer is unchanged in the full complex (Supplementary Fig. 5a). CSPs were observed for residues on the surface opposite to that binding the histones, which result from the previously reported interaction with the Rtt109 C-terminal region^34^. The SANS curve of the Asf1–H3^35–135^:H4–Rtt109–Vps75_2_ complex acquired in 100% D_2_O buffer, using ^1^H-Asf1–H3^35–135^:H4 and ^2^H(70%)-Rtt109–Vps75, reported on the conformation of the Asf1–H3:H4 sub-complex within the full complex. The curve fitted well to that predicted from the Asf1–H3:H4 structure of PDB entry 2hue in the context of the full complex model (Supplementary Fig. 5b), confirming that this Asf1–H3:H4 structure can be used as a semi-flexible building block in the M3 protocol.

The crystal structures available for Vps75_2_ display considerable variability, originating from a kink in the middle of the Vps75_2_ coiled-coil domain (Supplementary Fig. 5c). To determine the conformation of the Rtt109–Vps75_2_ sub-complex within Asf1–H3:H4–Rtt109–Vps75_2_, we performed a 400-ns molecular dynamics (MD) simulation starting from the crystal structure of Rtt109–Vps75_2_ (PDB entry 3q66) and separated the snapshots into clusters according to the orientation of Vps75(A) relative to Rtt109. We then scored the snapshots with respect to the normalized consensus χ^2^ (Methods) between their back-calculated SANS curves and the experimental curves, measured in 42%:58% D_2_O:H_2_O buffer for the Asf1–H3:H4–Rtt109–Vps75_2_^1–225^ complex containing either ^2^H-Vps75^1–225^ or ^2^H-Rtt109/^2^H-Vps75^1–225^ (Supplementary Fig. 5d–e). These curves reported on the conformation of Vps75_2_ and Rtt109–Vps75_2_, respectively, in the context of the Asf1–H3:H4–Rtt109–Vps75_2_^1–225^ complex. We selected the 10 structures with the lowest normalized consensus χ^2^ to use as semi-flexible building blocks for ensemble docking in M3 (Supplementary Fig. 5d). The fit of each of the 10 selected conformations to the SANS data were much-improved relative to that of PDB entry 3q66 (Supplementary Fig. 5e). None of the selected conformations, which belonged to three different clusters (Supplementary Fig. 5d), had been observed before; all display a rotation of Vps75(A) relative to Rtt109, which results in much-improved substrate accessibility (Supplementary Fig. 5f).

The M3 protocol incorporated a total of 145 PRE-derived distance restraints and six SANS curves. At the end of the protocol the structures were separated into clusters (Supplementary Fig. 6) and scored as described in Methods according to the protocol of Karaca et al.^31^. Structures from the best-scoring cluster with a fit to the experimental data better than any structure of the second-best-scoring cluster were selected as members of the final structural ensemble (Supplementary Fig. 6, Methods). The resulting final ensemble of 33 structures shows an average pairwise backbone RMSD (root-mean-square deviation) of 2.0 Å and an average RMSD to the ensemble center of 1.4 Å, demonstrating that the Asf1–H3:H4–Rtt109–Vps75_2_ structural model is defined to high precision by the experimental data.

The structure closest to the ensemble center is shown in Fig. 1. The complex adopts a doughnut-like structure with the curved and twisted Vps75 homodimer bridging between Rtt109 and Asf1–H3:H4 (Fig. 1). The doughnut is closed on the opposite side to Vps75 by the Rtt109 C-terminal tail contacting Asf1.

**Fig. 2:**
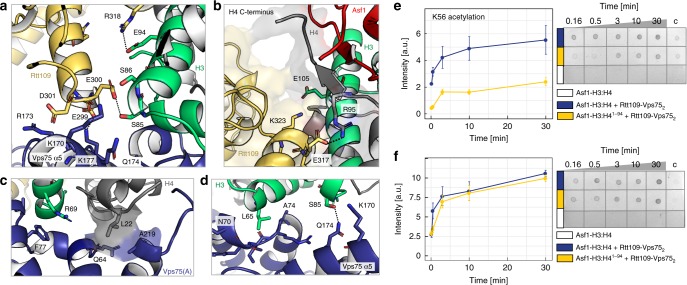
K9-acetylation does not depend on the H4 C-terminal region. **a** The interface of H3, Rtt109 and Vps75(A) displays a network of hydrogen bonds (dashed lines) and electrostatic contacts. **b** Interface between the H4 C-terminal region and Rtt109. **c**, **d** Interface of H3:H4 and Vps75(A): hydrophobic contacts involve H4-L22, H3-R69 and L65, Vps75-F77, Q64, A219 and the backbone of stretch 70–74; electrostatic contacts involve Vps75-K170 and Q174 and H3-S85. **e**, **f** Time-courses (left) of K56- and K9-acetylation quantified by dot-blot assays (right). The H4 95–102 stretch is important for K56ac (**e**) but not for K9ac (**f**). Experimental conditions: 0.2 μM Rtt109–Vps75_2_, 0.2 μM Asf1−H3:H4, 2 μM Ac-CoA, 10 mM HEPES pH 8.0, 100 mM NaCl. Because the control reaction with 0.2 μM Asf1−H3:H4 (white) did not give signal above the background, an additional negative control with 6 μM Asf1−H3:H4 (column “c”) was used for normalization and comparison of the experimental repeats. The data were averaged over four experiments; the error bars are the standard errors of the means. Source data are provided as a Source Data file

In the *Sc* Asf1–H3:H4–Rtt109–Vps75_2_ complex, Rtt109 interacts with the H3-α2 helix in a similar manner to that seen in the *Af* Asf1–H3:H4–Rtt109 complex^24^ (Fig. 2a). H3-E94 and E105 are engaged in ionic interactions with Rtt109-R318 and K323, respectively; accordingly, attachment of a paramagnetic tag to the mutant H3-E94C severely disturbed complex formation (Supplementary Fig. 7a). Rtt109-E300 forms close electrostatic contacts with H3-S85 and S86, while Rtt109-E299 and D301 interact with the positively charged Vps75(A) α5 helix (Fig. 2d), thus rationalizing previous functional data that demonstrated the importance of the Rtt109-^299^EED^301^ stretch for both H3-K9ac and H3-K27ac in vivo^18^.

Rtt109 also contacts H4; H4-R95 forms a salt bridge with Rtt109-E317 (Fig. 2b) and the NMR signals of H4-G102 disappear upon complex formation (Supplementary Fig. 7b). Similar to the *Af* Asf1–H3:H4–Rtt109 complex^24^, we found that H3-K56ac was impaired by deletion of the last 8 residues (95–102) of H4 in the Asf1–H3:H4–Rtt109–Vps75_2_ complex (Fig. 2e). However, the same deletion had only a moderate effect on H3-K9ac (Fig. 2f), indicating that, in contrast to K56ac, K9ac does not depend on the contacts between the H4 C-terminal residues and Rtt109.

Vps75(A) recognizes the H3:H4 dimer through both hydrophobic and electrostatic contacts involving H3-α1 and H4-α1 (Fig. 2c, d). H3-R69 stacks on Vps75(A)-F77 (Fig. 2c), while H3-L65 contacts the backbone of the Vps75(A) loop 70–73 (Fig. 2d). Accordingly, the H3-R69E/R72E double mutation weakened complex formation (Supplementary Fig. 7c). H4-L22 fits snugly in a small pocket formed by Vps75(A)-Q64 and A219 (Fig. 2c). H3-S85 engages in a hydrogen bond and electrostatic contacts with Q174 and K170 of Vps75(A)-α5 (Fig. 2d). Thus, in contrast to earlier predictions^18^, the Vps75 acidic patch centered around ^206^EE^207^ is not involved in the recognition of the histone core but rather builds an accessible acidic surface within the central cavity of the doughnut (Fig. 1c).

**Fig. 3:**
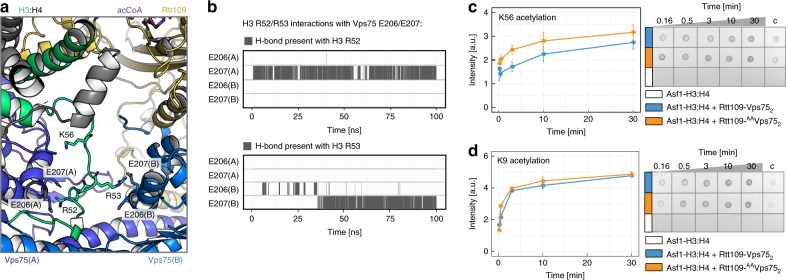
The Vps75 acidic patch keeps K56 away from the Rtt109 catalytic pocket. **a** Final snapshot of the 100-ns MD trajectory of Asf1–H3^35–135^:H4–Rtt109–Vps75_2_^1–225^, starting from the structure of Fig. 1. The stretch H3^35–59^ was initially built in an extended conformation (Supplementary Fig. 8). During the MD run, stable H-bonds were formed between H3-R52 and Vps75(A)-E207 and between H3-R53 and Vps75(B)-E207; these contacts are incompatible with H3-K56 binding in the Rtt109 catalytic center. **b** Analysis of the H-bonds during the MD run. **c**, **d** Time-courses (left) of K56- and K9-acetylation quantified by dot-blot assays (right). The assays and data-analysis were done as described in the legend to Fig. 2. Source data are provided as a Source Data file

We next investigated whether the Vps75 acidic patch, while not contacting the histone core, interacts with the basic amino acids located before H3-α1. Indications of this interaction appeared in the ^1^H-^13^C NMR spectra of Vps75 in both the Asf1–H3:H4–Rtt109–Vps75_2_ and Asf1–H3^35–135^:H4–Rtt109–Vps75_2_ complexes, in which Vps75-L203 and I208, located around the ^206^EE^207^ dyad, displayed considerable CSPs relative to Rtt109–Vps75_2_, despite not being in contact with the histone core (Supplementary Fig. 1d–e). To reveal the molecular basis of these CSPs, we performed molecular dynamics (MD) simulations of the Asf1–H3^35–135^:H4–Rtt109–Vps75_2_^1–225^ complex, which includes 25 amino acids of the H3 N-terminal domain (35–59), and observed the formation of stable contacts between Vps75-^206^EE^207^ and H3-^53^RR^54^ (Fig. 3, Supplementary Fig. 8). These contacts lock H3-K56 in a position far from the Rtt109 catalytic site and would act to reduce H3-K56ac, thereby favoring acetylation of the lysine residues in the N-terminal tail. In agreement with this hypothesis, we found that the Asf1–H3:H4–Rtt109–^AA^Vps75_2_ complex, assembled with the Vps75-E206A/E207A double mutant (^AA^Vps75), acetylates K56 more efficiently than the wild-type complex (Fig. 3).

### The Vps75 C-terminal tail guides substrate selection

**Fig. 4:**
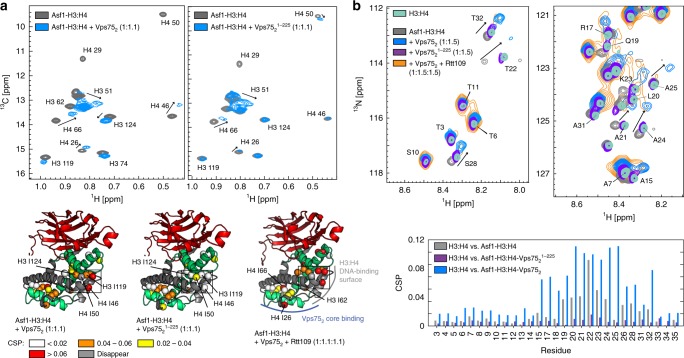
The Vps75 CTAD interacts with both the H3 core and tail. **a** Comparison of the ^1^H-^13^C HMQC spectra of ILV methyl-labeled H3:H4 in the Asf1–H3:H4 complex and upon addition of either 1.1 equivalents of Vps75_2_ (left) or Vps75_2_^1–225^ (right). The corresponding CSPs mapped on the Asf1–H3:H4 structure (PDB entry 2hue) are shown in the bottom-left and bottom-middle panel, respectively. The CSPs obtained after addition of 1.1 equivalents of Rtt109–Vps75_2_ to Asf1–H3:H4 (bottom-right panel) are very similar to those obtained with Vps75_2_ and demonstrate that the Vps75 CTAD interacts with the DNA-binding surface of H3:H4 in both Asf1–H3:H4–Vps75_2_ and Asf1–H3:H4–Rtt109–Vps75_2_. **b** Top, ^1^H-^15^N HSQC spectra of H3 in the H3:H4 dimer, in the Asf1–H3:H4 complex and upon further addition of Vps75_2_, Vps75_2_^1–225^ or Rtt109–Vps75_2_. Bottom, the corresponding CSPs plot. In Asf1–H3:H4, the H3 tail interacts with Asf1, as reported by Lercher et al.^34^. In the presence of Vps75_2_^1–225^, the H3 tail is released from Asf1 and recovers the CSs of the H3:H4 dimer; in the presence of Vps75_2_, the H3 tail peaks move to new positions. The NMR experimental conditions were as in Fig. 1. Source data are provided as a Source Data file

In the structure of Asf1–H3:H4–Rtt109–Vps75_2_, the active site of Rtt109, which faces the central cavity of the doughnut, is juxtaposed to the basic DNA-binding surface of H3:H4, while the acidic surface of the Vps75 dimer forms the floor of the cavity (Fig. 1c). The positively charged DNA-binding surface builds an electrostatic barrier to the basic H3 tail, which needs to enter the cavity in order to reach the Rtt109 active site. NMR CSPs of the ^1^H,^13^C-labeled methyl groups of H3:H4 in the complex with full-length Vps75 (Asf1–H3:H4–Rtt109–Vps75_2_) relative to the complex assembled with C-terminally truncated Vps75 (Asf1–H3:H4–Rtt109–Vps75_2_^1–225^) reveal that the Vps75 CTAD interacts with the H3:H4 DNA-binding surface (Fig. 4a), generating an electrostatic environment that allows the positively charged H3 tail to enter the cavity.

**Fig. 5:**
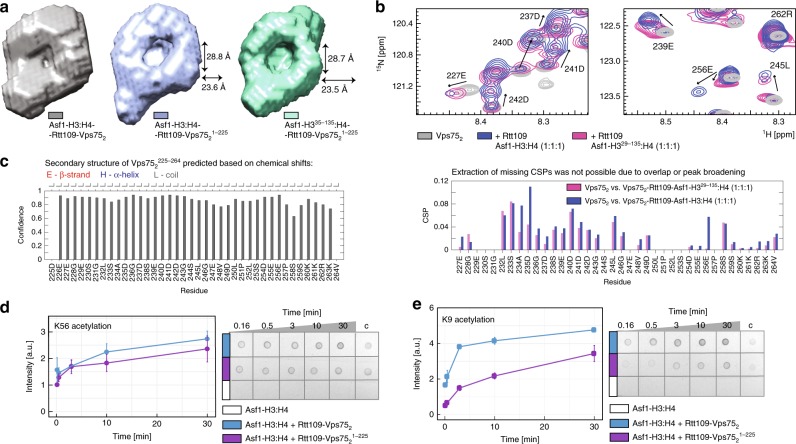
The disordered Vps75 CTAD guides substrate specificity. **a** The SANS-derived *ab initio* envelopes of Asf1–H3:H4–Rtt109–Vps75_2_^1–225^ (middle) and Asf1–H3^35–135^:H4–Rtt109–Vps75_2_^1–225^ (right) display a central empty cavity, which is filled by scattering units in the complex containing full-length proteins (left). Therefore the H3 tail does not enter the cavity in the absence of the Vps75 CTAD. **b** Overlay of ^1^H-^15^N spectra of Vps75_2_ in isolation and as part of either Asf1–H3:H4–Rtt109–Vps75_2_ or Asf1–H3^29–135^:H4–Rtt109–Vps75_2_. Only signals of the Vps75 CTAD are visible. In Asf1–H3:H4–Rtt109–Vps75_2_, the CTADs of Vps75(A) and Vps75(B) display different chemical shifts: the peaks of one of the two CTADs remain in the same positions as those from Vps75_2_, while those of the other CTAD move to new positions. The peaks of residues 234–246 move less far in Asf1–H3^35–135^:H4–Rtt109–Vps75_2_ (CSP plot, bottom). **c** TALOS-N CS analysis of Vps75^226–264^ in the context of full-length Vps75_2_ shows that the Vps75 tail is disordered. The same narrow CS dispersion is observed in the full complex (Supplementary Fig. 9). **d**, **e** Time-courses (left) of K56- (**d**) and K9-acetylation (**e**) quantified by dot-blot assays (right). The assays and data-analysis were done as described in the legend to Fig. 2. Source data are provided as a Source Data file

Next, we tested whether the Vps75 CTAD interacts directly with the H3 tail. We compared the 2D ^1^H-^15^N NMR spectrum of H3 in the free histone dimer H3:H4 with those of H3 in the Asf1–H3:H4 complex and after addition of one equivalent of either Vps75_2_ or Vps75_2_^1–225^. In the presence of Vps75_2_, the chemical shifts of H3 residues 7–37 differed from those measured in either H3:H4 or Asf1–H3:H4, while in the presence of the C-terminally truncated Vps75_2_^1–225^, the chemical shifts of the H3 tail were identical to those of the free histone dimer (Fig. 4b). This result demonstrates that the H3 tail interacts with the Vps75 CTAD. Upon addition of Rtt109 to the Asf1–H3:H4–Vps75_2_ complex, the positions of the H3 tail ^1^H-^15^N peaks were unchanged, but their intensity decreased. We reasoned that this intensity reduction is a consequence of the H3 tail becoming confined to the cavity of the doughnut in the full complex, which leads to some loss of mobility. To verify this hypothesis, we compared the *ab initio* models obtained from the SANS curves of the Asf1–H3:H4–Rtt109–Vps75_2_ and Asf1–H3^35–135^:H4–Rtt109–Vps75_2_^1–225^ complexes (Fig. 5a). The 25-Å-wide cavity in the middle of the doughnut, which is apparent in the envelope of Asf1–H3^35–135^:H4–Rtt109–Vps75_2_^1–225^, is filled with scattering units in the envelope of Asf1–H3:H4–Rtt109–Vps75_2_, suggesting that both the Vps75 CTAD and the H3 N-terminal tail occupy this cavity in the full complex. We conclude that upon binding the H3 tail, the Vps75 CTAD attracts it to the central cavity of the complex, close to the Rtt109 active site. In agreement with this conclusion, the *ab initio* model of the Asf1–H3:H4–Rtt109–Vps75_2_^1–225^ complex, containing full-length H3 but C-terminally truncated Vps75, showed a cavity of the same size as that in the Asf1–H3^35–135^:H4–Rtt109–Vps75_2_^1–225^ complex (Fig. 5a), confirming that in the absence of the Vps75 CTAD, the H3 tail is not localized to the center of the doughnut.

To further verify that the Vps75 CTAD binds both the DNA-binding surface of the H3:H4 dimer and the H3 tail, we monitored the 2D ^1^H-^15^N NMR spectrum of the Vps75 CTAD (Fig. 5b). The CTADs of the two Vps75 subunits in the Asf1–H3:H4–Rtt109–Vps75_2_ complex displayed different chemical shifts: the peaks of the C-terminal residues of one Vps75 subunit remained in the same positions as those of the free Vps75 dimer, while many of the corresponding peaks of the second subunit moved to new positions (Fig. 5b). The CSPs were concentrated in the 233–259 stretch, containing 11 of the 14 negatively charged residues of the Vps75 CTAD. These shifts are not a consequence of binding Rtt109 (Supplementary Fig. 9), and therefore indicate that the region 233–259 of one of the two Vps75 subunits binds the Asf1–H3:H4 sub-complex. When we compared the ^1^H-^15^N CSPs of the Vps75 CTAD in the Asf1–H3:H4–Rtt109–Vps75_2_ and the Asf1–H3^29–135^:H4–Rtt109–Vps75_2_ complexes (relative to free Vps75_2_), we observed that the CSPs of residues 234–246 in the complex assembled with N-terminally truncated H3 were smaller than in that assembled with full-length H3 (Fig. 5b), confirming the interaction between the Vps75 CTAD and the H3 tail. Despite binding to each other, both the H3 N-terminal domain and the Vps75 CTAD remain disordered (Figs. 4b, 5c and Supplementary Fig. 9), hence conserving a substantial portion of their free-form conformational entropy. In agreement with the structural data, the removal of the Vps75 CTAD severely impacts H3-K9ac, but has no effect on H3-K56ac (Fig. 5d, e). Fittingly, the Vps75 CTAD is conserved in species with Rtt109-dependent histone acetylation (Supplementary Fig. 2) and its deletion in vivo results in high vulnerability to genotoxins, comparable to the *VPS75* deletion strain^35^.

## Discussion

**Fig. 6:**
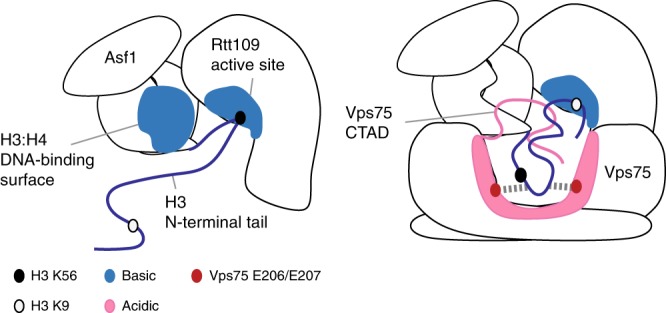
Fuzzy electrostatic interactions promote acetylation of lysine residues in the H3 tail. Left, the mechanism of chaperoning H3-K56 to the Rtt109 catalytic pocket is based on well-known enzyme-recruitment and substrate-presentation processes. Right, the mechanism by which Vps75 chaperones lysine residues in the H3 tail to the Rtt109 catalytic pocket differs from the canonical substrate-presentation process and includes confinement of the H3 tail in the proximity of the Rtt109 catalytic pocket via fuzzy electrostatic interactions occurring between two disordered protein domains, the Vps75 CTAD and the H3 tail.

The histone acetyltransferase Rtt109 acetylates multiple substrates located either close to structured elements (H3-K56) or within intrinsically disordered regions (H3-K9, K23, K27). Histone chaperones act as promoters of enzyme activity and specificity switches. The mechanisms by which chaperones promote chemical modification of structured regions have been described in many systems and include binding to both enzyme and substrate, optimization of interaction interfaces and substrate presentation. In the case of histone acetylation, the recently reported structure of the *Af* Asf1–H3:H4–Rtt109 complex demonstrates that Asf1 promotes the modification of H3-K56, located just before the start of the H3 core, by stabilizing the structure of the C-terminal region of H4 for optimal interaction of the H3:H4 dimer with Rtt109^24^. Conversely, the mechanisms by which chaperones help localizing modification sites within long disordered regions to a well-defined enzymatic pocket are not understood. Here, we demonstrate that Vps75 and Asf1 act in concert to promote acetylation of H3-K9, located in the long unstructured H3 tail, by a two-fold mechanism that minimizes the inevitable entropic costs associated with enzymatic modification of sites within intrinsically disordered regions (Fig. 6). First, Asf1 and Vps75 build — together with the substrate and the enzyme — a doughnut-shaped complex, whose central cavity is faced by the enzyme catalytic pocket. Second, one of the two negatively charged and disordered CTADs of the Vps75 dimer, which is attracted to the central cavity of the complex by the positively charged DNA-binding surface of the H3:H4 core, engages in fuzzy electrostatic interactions with the H3 tail, guiding it into the cavity and close to the Rtt109 catalytic pocket. The entropic cost associated with the confinement of the disordered tails of both Vps75 and H3 to the doughnut cavity could be compensated by the release of water molecules from the highly charged cavity and by the favorable electrostatic contacts of the Vps75 CTAD with both the H3:H4 core and the H3 tail. Finally, a structured, negatively charged region centered at Vps75-^206^EE^207^ assists the switch of acetylation specificity from K56 to sites in the H3 tail by recruiting H3-^53^RR^54^, acting to keep K56 away from the Rtt109 catalytic pocket.

Importantly, the positions and the line-widths of the ^1^H-^15^N NMR peaks of the Vps75 CTAD and the H3 tail demonstrate that, despite being localized to the cavity, they both remain disordered. Recently, an electrostatically-driven complex has been reported to form between the disordered portion of histone H1 and the intrinsically disordered protein ProTα^36,37^; the fast dissociation rate constant found for this fuzzy complex has been proposed to be common to interactions of this kind and important for rapid responses in regulatory processes. In the Asf1–H3:H4–Rtt109–Vps75_2_ complex, the disordered, fuzzy nature of the interaction between the Vps75 CTAD and the H3 tail may serve to facilitate the presentation of multiple lysine sites (K9, K23, and K27) to the Rtt109 catalytic site^12,26^. The mechanism of substrate localization described here uniquely supports the combination of specificity (acetylation of lysine residues within the H3 tail) and promiscuity (acetylation of multiple sites) that is necessary for cell function.

Many histone chaperones, such as CAF-1, Spt6, Daxx and all chaperones of the NAP-1 family, possess negatively charged disordered domains. While Rtt109 is the only histone-modifying enzyme whose dependence on chaperones has been studied in molecular detail, histone chaperones have been linked to deposition and removal of PTMs in many other chromatin-related processes^38^. This fact, together with the large number of disordered domains contained in the human proteome, suggest that interactions between unstructured sequences of the kind described here may represent a general mechanism of chaperoning disordered protein domains in cell processes, including and beyond epigenetics.

## Methods

### Protein expression and purification

Plasmids for *Xenopus laevis* histone H3/H4 (pET3d/a), *S. cerevisiae* Vps75 (pET28a) and *S. cerevisiae* Asf1 (2–169, pGEX6P-2) were a kind gift from the Luger laboratory (University of Colorado Boulder).

Asf1^2–169^ (UniProtKB accession code P32447), Rtt109 (Q07794) and Vps75 (P53853) were cloned into pETM11, containing an N-terminal hexa-histidine tag, followed by a TEV protease cleavage site. For each protein, the plasmid was transformed into *E. coli* BL21(DE3) and expression was induced by addition of 0.5 mM IPTG at OD_600_ = 0.6–0.8 and continued for 14–16 h at 16 °C. Cells were lysed by sonication in the wash buffer with the addition of 1× protease inhibitors cocktail (Roche; 11697498001).

Vps75 was purified by immobilized-metal-ion affinity chromatography (IMAC) (GE Healthcare Histrap HP 5 ml). The column was equilibrated and washed with wash buffer (25 mM Tris–HCl pH 7.5, 500 mM NaCl, 5 mM BME, 20 mM imidazole) and eluted with a gradient to 100% elution buffer (25 mM Tris–HCl pH 7.5, 500 mM NaCl, 5 mM BME, 400 mM imidazole) over 20 CV (column volumes). The tagged protein was then incubated with TEV protease overnight at 4 °C. To isolate the cleaved protein, the mixture was injected onto a Histrap HP column equilibrated in wash buffer and the flow-through was collected. Cleaved Vps75 was further purified by ion-exchange (GE Healthcare Hi-trap Q, 25 mM Tris–HCl pH 7.5, 5 mM BME, gradient from 0.15 to 1 M NaCl over 20 CV) and size-exclusion chromatography (Superdex S200 16/60; 25 mM Tris–HCl pH 7.5, 500 mM NaCl, 5 mM BME). Asf1 was purified identically to Vps75, except for a different imidazole gradient (10 mM–500 mM over 15 CV). Rtt109 was purified identically to Vps75, except for the ion-exchange step, which was performed with GE Healthcare Heparin HP 5 ml, in 25 mM Tris–HCl pH 7.5, 5 mM BME, gradient from 0.3 to 1 M NaCl over 15 CV. All steps of the Rtt109 purification were done at 4 °C.

Histones H3 (P84233) and H4 (P62799) were expressed and purified as described by Luger et al.^39^. Briefly, the pET3d plasmids containing either H3 or H4 were transformed into *E. coli* BL21(DE3)pLysS cells. Expression was induced at OD_600_ = 0.6 with addition of 0.2 mM IPTG and continued for the next 2.5 h at 37 °C. The cells were harvested by centrifugation, resuspended in wash buffer (50 mM Tris–HCl, pH 7.5, 100 mM NaC1, 1 mM Na-EDTA, 5 mM BME) and lysed by sonication. The pellet containing inclusion bodies was washed twice with wash buffer with 1% (v/v) Triton X-100 and twice with wash buffer. The pellet was solubilized in unfolding buffer (7 M guanidinium hydrochloride, 20 mM Tris–HCl, pH 7.5, 10 mM DTT). The histones were then purified by SEC (Superdex S200 16/60; 7 M deionized urea, 20 mM sodium acetate, pH 5.2, 1 M NaCl, 5 mM BME, 1 mM Na-EDTA) and ion-exchange (GE Healthcare HiTrap SP HP, 7 M deionized urea, 20 mM sodium acetate, pH 5.2, 5 mM 2-mercaptoethanol, 1 mM Na-EDTA, gradient from 0.2 M to 1 M NaCl over 15 CV). All purification steps were done at 4 °C; purified histones were either used for reconstitution immediately or flash-frozen and stored at −80 °C.

Protein mutants were generated following the QuikChange-XL protocol (Agilent Technologies).

Perdeuterated and 70% deuterated proteins for SANS measurements were expressed in 100% D_2_O M9 minimal medium supplemented with ^2^H-glycerol or ^1^H-glucose, respectively. ^15^N-labeled histones were produced in H_2_O M9 minimal medium supplemented with ^15^NH_4_Cl. ILV methyl-labeled proteins were expressed in 100% D_2_O M9 minimal medium supplemented with α-ketobutyric acid sodium salt (60 mg/L, Sigma, cat. no. 589276) and α-ketoisovaleric acid sodium salt (120 mg/mL, Sigma, cat. no. 691887). Pro-S LV methyl labeling was achieved with the NMR-Bio precursor (DLAM-LV^proS^ kit).

Labeled and mutant proteins were purified identically to the unlabeled wild-type proteins.

### Complex assembly

The H3 and H4 histones were mixed in an equimolar ratio in 7 M urea, 10 mM Tris–HCl pH 7.5, 1 M NaCl, 1 mM Na-EDTA, 5 mM BME and dialysed at 4 °C into 10 mM Tris–HCl pH 7.5, 2 M NaCl, 1 mM Na-EDTA, 5 mM BME. The refolded H3:H4 dimer was separated by size-exclusion chromatography (SEC) in the same buffer. The Asf1–H3:H4 sub-complex was prepared by mixing the H3:H4 dimer with Asf1 in a 1:1.1 ratio in 50 mM Tris–HCl pH 7.5, 0.5 M NaCl, 5 mM BME followed by SEC. The Rtt109–Vps75_2_ sub-complex was reconstituted with a 1.1:1 Rtt109:Vps75_2_ ratio in the NMR/SANS buffer (50 mM citrate pH 6.5, 150 mM NaCl, 5 mM BME) and subsequently purified by SEC. The full complex was assembled by mixing the two sub-complexes in a 1:1 ratio followed by SEC in the NMR/SANS buffer. All size-exclusion chromatography runs were performed with a Superdex S200 10/300 column.

### NMR data collection and processing

All NMR spectra were recorded at 298 K (unless specified otherwise) on Bruker Avance III HD 600 MHz and 850 MHz spectrometers equipped with nitrogen-cooled and helium-cooled cryogenic inverse HCN probe-heads, respectively. NMR data were processed with the NMRPipe^40^ or Topspin (Bruker) software packages and analyzed within CcpNmr Analysis v2.4^41^. Line-shape fitting was performed with FuDA (https://www.ucl.ac.uk/hansen-lab/fuda/). Sampling schedules for data sets recorded with non-uniform sampling (NUS) were created using the nus@HMS online schedule-generator (http://gwagner.med.harvard.edu/intranet/hmsIST/gensched_new.html). Time-domain data matrices for data sets recorded with NUS were reconstructed prior to Fourier transformation using iterative soft-thresholding as implemented in the hmsIST program^42^.

### Two-dimensional correlation spectra

2D ^1^H-^15^N spectra were recorded using either ^15^N-HSQC^43^ or ^15^N-TROSY-HSQC pulse sequences^44^. 2D ^1^H-^13^C spectra were recorded using the ^13^C-HMQC (methyl-TROSY) pulse-sequence^45^. Chemical shift perturbations (CSPs) were calculated according to

1 $$d = \root {2} \of {{\frac{1}{2}\left( {\Delta \delta _{\mathrm{H}}^2 + \left( {0.15 \cdot \Delta \delta _{\mathrm{N}}} \right)^2} \right)}}\,{\mathrm{or}}\,d = \root {2} \of {{\frac{1}{2}\left( {\Delta \delta _{\mathrm{H}}^2 + \left( {0.3 \cdot \Delta \delta _{\mathrm{C}}} \right)^2} \right)}}$$

### Assignment experiments

Experiments for the assignment of Vps75 were performed with isolated ^2^H,^13^C,^15^N-ILV-methyl-protonated Vps75^1–225^ at 0.8 mM protein concentration in 20 mM Tris–HCl pH 7.5, 200 mM NaCl, 1 mM DTT at 300 K. 56% of backbone amide resonances were assigned using TROSY-HNCA, TROSY-HNCO, TROSY-HN(CA)CO, TROSY-HN(CO)CA and TROSY-HNCACB experiments^46^ (Supplementary Fig. 10). A subset of the ILV methyl resonances (I28, I54, I60, I127, I138, I155, I160, I179, V121, L16 & L223) were assigned through Ile,Leu-(HM)CM(CGCBCA)NH and Val-(HM)CM(CBCA)NH experiments^47^. Methyl resonances of I28, I54, I72, I127, V25, V32, V61, V124, V213 & L188 were assigned or verified via point mutations. The remaining methyl resonances were assigned using a 4D HCCH HMQC-NOESY-HMQC spectrum recorded with NUS^48^ in combination with the predicted NOEs from the PDB entry 2zd7^35^. Stereospecific assignment of the L and V methyl resonances was achieved using pro-S LV-labeling (Supplementary Fig. 11). In total, we assigned 17/17 Ile, 10/13 Leu, and 10/10 Val residues.

The ILV methyl-group assignments were then transferred from Vps75^1–225^ to the free full-length Vps75 in 50 mM citrate pH 6.5, 150 mM NaCl, 5 mM BME and thence to the Vps75 in the full complex following the CSPs in Vps75 titrations with Asf1–H3:H4 or Rtt109 (Supplementary Fig. 11). The final assignment was verified by pairing diastereotopic methyl-groups of leucine and valine residues with the assistance of a 3D experiment in which the ^1^H and ^13^C resonances of the methyl groups were correlated with the ^13^C resonances of the directly bonded methine carbon (Cγ and Cβ for leucine and valine residues, respectively), thereby allowing methyl-pairs to be identified from their common methine resonance. The pulse-sequence for this experiment comprises an out-and-back magnetization-transfer-pathway starting and ending on the methyl protons, using COSY-type transfers between the methyl and methine carbons and constant-time chemical-shift evolution periods for both indirect ^13^C dimensions.

The backbone amide resonances of the Vps75 CTAD were assigned with HNCO, HNCACB and HN(CO)CACB experiments^49–51^ recorded on ^13^C,^15^N-labeled full-length Vps75 at 0.6 mM in 50 mM citrate pH 5.6, 150 mM NaCl, 5 mM BME (Supplementary Fig. 9).

Isoleucine methyl resonances of Rtt109 were assigned with a 3D (H)CCH HMQC-NOESY-HMQC spectrum recorded on perdeuterated, ^13^C,^1^H-methyl-I-labeled Rtt109 at ~0.6 mM in complex with perdeuterated Vps75 in 100% D_2_O NMR/SANS buffer. Experimental NOEs were compared to those predicted from PDB entry 3q66^29^. In total, we could assign 9/31 Ile δ-methyl group resonances.

H3:H4 assignment experiments were recorded with 0.66 mM ILV methyl-labeled H3:H4 in complex with perdeuterated Asf1 in 20 mM Tris–HCl pH 7.5, 1 M NaCl, 1 mM Na-EDTA, 5 mM BME in 100% D_2_O. The methyl groups were assigned by means of 3D (H)CCH- HMQC-NOESY-HMQC and 3D HCH HMQC-NOESY^52,53^ spectra in combination with the NOEs predicted from PDB entry 2hue^21^ (Supplementary Fig. 11). In addition, methyl resonances belonging to H4 were identified from a ^13^C-HMQC spectrum of ILV methyl-labeled H4 in the context of the Asf1–H3:H4 complex. In total, we assigned 5/7 Ile, 4/12 Leu and 1/6 Val residues of H3 and 6/6 Ile, 1/8 Leu and 4/9 Val residues of H4.

The Asf1 assignment was described by Lercher et al.^34^. Secondary-structure analysis of the Vps75 CTAD was performed with TALOS-N^54^.

### Paramagnetic relaxation enhancement experiments (PREs)

PRE data-sets were acquired for eight paramagnetic centers engineered at different positions on the H3:H4 dimer to measure distance-restraints to Vps75- or Rtt109-methyl groups in the Asf1–H3:H4–Rtt109–Vps75_2_ complex (Supplementary Fig. 3a).

Single-cysteine mutations were introduced in H4 or in H3-C110A, where the wild-type cysteine was substituted by alanine. The histones were then purified and refolded as the wild-type proteins, followed by SEC in the refolding buffer without reducing agent (10 mM Tris–HCl pH 7.5, 2 M NaCl). The paramagnetic agent (3-(2-iodoacetoamido)-PROXYL radical (Sigma-Aldrich, cat. no. 253421) was added to the H3(C110A):H4 dimer, containing the single cysteine mutation, immediately after the SEC step. The coupling between the paramagnetic agent and the free cysteine was allowed to proceed overnight at 4 °C in the dark.

The unreacted paramagnetic agent was removed during an additional SEC step in 10 mM Tris–HCl pH 7.5, 2 M NaCl, after which the H3(C110A):H4 dimers, containing the paramagnetic center, were used for reconstitution of the complex. Rtt109, Vps75, and Asf1 were exchanged into the NMR buffer with no reducing agent using a HiPrep 26/10 desalting column (GE Healthcare). The complexes were reconstituted and measured in the 99% D_2_O NMR buffer without BME. The sample concentrations varied between 30 and 90 μM.

^1^H-^13^C HMQC spectra were acquired for the paramagnetic and diamagnetic (reduced with 2 mM ascorbic acid) states of the complexes. In order to derive the peak intensity in the paramagnetic (*I*^para^) and the diamagnetic (*I*^dia^) states, the peaks were fitted with FuDA assuming Lorentzian line-shapes (spectra were processed using exponential apodization in both dimensions). Overlapped peaks were fitted as groups. Only well-resolved peaks unambiguously corresponding to Vps75(A) or Vps75(B) were used to quantify distances between H3:H4 and ILV methyl-labeled Vps75. Additional peaks were fitted for the data-sets recorded with H3-Q76C and H4-T82C, where preliminary structural calculations indicated that the paramagnetic tags are far away from the Vps75(B) earmuff domain. This allowed us to select five additional peaks, for which we assumed that only one of the two overlapped resonances of Vps75(A) and Vps75(B) is affected by the paramagnetic center, while the (*I*^para^/*I*^dia^) height ratio of the portion of the peak corresponding to Vps75(B) is equal to 1.

The fitted volumes and line-widths from FuDA were converted into peak-heights using:

2 $$I = \frac{V}{{LW_{\mathrm{H}} \cdot LW_{\mathrm{C}}}}$$

where *I* is the peak height, *V* is the peak volume and *LW*_H_ and *LW*_C_ are the fitted line-widths in the ^1^H and ^13^C dimensions, respectively. The peak-height ratios (*I*^para^/*I*^dia^) were calculated for each data set and the experimental errors for each *I*^para^/*I*^dia^ ratio were derived by the error propagation rules from the standard deviation of the noise in the HMQC spectra.

Diamagnetic transverse relaxation rates for the ^1^H single-quantum coherence (*R*_2_^diaH^) and the ^1^H-^13^C double-quantum coherence (*R*_2_^diaHC^) were quantified using the pulse schemes reported in Korzhnev et al.^55^ and Tugarinov et al.^56,57^. Relaxation delays for the quantification of *R*_2_^diaHC^ of both Vps75 and Rtt109 methyl resonances were 0, 2, 4, 7, 10, 13 & 20 ms. Relaxation delays for the quantification of *R*_2_^diaH^ were 0, 2, 4, 6, 8, 10, 13 & 16 ms for Vps75 resonances, and 0, 3, 6, 9, 12, 16 & 20 ms for Rtt109 resonances. The peak-heights were fitted to a mono-exponential decay function to extract *R*_2_^diaH^ and *R*_2_^diaHC^.

The resulting *R*_2_^diaH^, *R*_2_^diaHC^ and the *I*^para^/*I*^dia^ values were used to calculate the transverse PRE rates (Γ_2_) according to:

3 $$\frac{{I^{{\mathrm{para}}}}}{{I^{{\mathrm{dia}}}}} = \frac{{exp( - {\mathrm{\Gamma }}_{2 \cdot }\tau _{{\mathrm{HMQC}}})R_2^{{\mathrm{diaH}}}R_2^{{\mathrm{diaHC}}}}}{{({\mathrm{\Gamma }}_2 + R_2^{{\mathrm{diaH}}})({\mathrm{\Gamma }}_2 + R_2^{{\mathrm{diaHC}}})}}$$

where *τ*_HMQC_ is the total duration of the constant-time delays in the ^13^C-HMQC pulse-sequence during which the magnetization is transverse on ^1^H (7.7 ms). The PRE rates were converted into distances using:

4 $$d = \root {6} \of {{\frac{K}{{{\mathrm{\Gamma }}_2}}\left( {4\tau _{\mathrm{c}} + \frac{{3\tau _{\mathrm{c}}}}{{\left( {1 + \omega ^2 \cdot \tau _{\mathrm{c}}^2} \right)}}} \right)}}$$

where *K* is a constant (1.233 × 10^–23^ cm^6^ s^−2^), ω is the proton Larmor frequency and *τ*_c_ is the correlation time of the electron–nucleus vector (81 ns). The correlation time was estimated by measuring the PRE values of Vps75 methyl groups in the presence of a paramagnetic tag on Vps75-E56C as part of the full complex. The measured PRE values were used as restraints for the simultaneous optimization of the tag conformations and *τ*_c_ via the PRE potential function in Xplor-NIH developed by Iwahara et al.^58^ using a protocol written by Nick Anthis. The minimization was run for 20 structures with the “obsig” setting for error-weighting of the PREs, which yielded a *τ*_c_ of 81 ± 7 ns.

The resulting distances were converted into M3 restraints as follows: the restraints were set between the Cβ of the tagged residues and the corresponding methyl groups; in order to account for the tag length and flexibility, an error of ± 6 Å was added to the experimental error; for *I*^para^/*I*^dia^ ratios below 0.2 the distance was set between 2.0 and 18.0 Å; for *I*^para^/I^dia^ ratios above 0.8 the distance was set between 24.0 and 99.0 Å.

### Structure calculation

The structural models were calculated with the M3 protocol^31^ employing HADDOCK 2.2. One building block comprised of the structure of the Asf1-H3:H4 sub-complex as in PDB entry 2hue (Asf1^1–164^, H3^60–134^, H4^20–101^). The second building block comprised of an ensemble of ten Rtt109–Vps75_2_ structures (Rtt109^1–418^, Vps75(A)^9–225^, Vps75(B)^2–231^). The Rtt109–Vps75_2_ conformers were generated by a 400-ns MD simulation (described below) and selected with respect to the normalized consensus χ^2^ between the calculated and experimental SANS curves of the complexes containing ^2^H-Vps75^1–225^ and ^2^H-Rtt109/^2^H-Vps75^1–225^. The docking was driven by 145 unique PRE-derived distance-restraints. An additional restraint was added to loosely limit the distance between the Rtt109-C terminus and Asf1, to ensure that the Rtt109 C-terminal tail can interact with Asf1, as reported in^34^.

The first docking stage (it0) was done with the default HADDOCK parameters; sampling of 180°-rotated molecules was disabled. As standard in HADDOCK, all residues were rigid at this stage. The resulting 5,000 structures, corresponding to 50,000 sampled conformers, were ranked using the restraint energy-based scoring, as described in ref. ^31^. In this case *E*_exp_ violation energy included one class of restraints, the PRE-derived distances. Only 15 structures with significantly low ln(*E*_exp_) values were identified as outliers in box-and-whisker plots with a whisker length of two times the IQR. Each of these 15 structures was submitted ten times to the second docking stage (it1). Side-chains of residues at the interfaces, as defined by HADDOCK based on the analysis of intermolecular contacts within a 5-Å cut-off, were flexible at this stage. The following parameters were changed to allow an extended search during it1: the temperature of the rigid-body torsion-angle-dynamics (TAD) search was increased to 5000 K; the number of steps was increased to 20,000; the number of rigid-body cooling steps was increased to 20,000; the factor time step of annealing was decreased to 4. After it1, 150 structures were scored against the SANS data (Supplementary Fig. 4) using CRYSON^59^. The χ^2^ values for different data sets were individually normalized:

5 $$\chi _{{\mathrm{norm}}}^2 = \frac{{\chi ^2 - \chi _{{\mathrm{min}}}^2}}{{\chi _{{\mathrm{max}}}^2 - \chi _{{\mathrm{min}}}^2}}$$

The fitness parameter represents the sum of normalized χ^2^ values and evaluates the overall fit of the structures to the SANS data. The structures were clustered by orientational RMSD (o-RMSD)^31^, calculated with respect to the structure with the best fitness. 33 members were selected from the best cluster (green in Supplementary Fig. 6) to represent the final ensemble; the selected structures had better fitness than any member of the second best cluster (blue in Supplementary Fig. 6). The structure with the lowest average RMSD (calculated on backbone atoms) to the 33 selected structures was chosen for further minimization in water (described below) and subsequent deposition to the PDB (entry 6o22).

### SANS data collection and analysis

Twelve damples with different combinations of uniformly perdeuterated, 70% deuterated and protonated proteins were measured in the SANS buffer containing 0%, 42% or 100% D_2_O (Supplementary Fig. 4c). The complex concentration varied between 16 and 36 μM (2.7 mg/mL and 6 mg/mL).

5 measurements (samples 1–5 in Supplementary Fig. 4c) were carried out at the KWS-1 beamline^60^ at the Juelich Centre for Neutron Sciences (JCNS outstation at MLZ, Garching, Germany). Two detector configurations of 1.5 m and 4 m were used for each sample with a collimation length of 4 m and a neutron wavelength of 5 Å. The remaining experiments were performed at the D22 instrument at the Institut Laue-Langevin (ILL, Grenoble, France) with a sample-detector distance of 4 m, collimation length of 4 m and a neutron wavelength of 6 Å.

All samples were measured at 298 K. Data reduction and radial integration were carried out according to the standard procedures with ILL and JCNS specific software. Buffer subtraction was done with PRIMUS^61^. Fits of the experimental data to the models were performed with CRYSON^59^ .

The *ab initio* models of the Asf1−H3:H4−Rtt109−Vps75_2_, Asf1−H3:H4−Rtt109−Vps75_2_^1–225^ and Asf1−H3^35–135^:H4−Rtt109−Vps75_2_^1–225^ complexes were generated with DAMMIF^62^. 20 independent *ab initio* models were calculated for each complex and averaged with DAMAVER. The averaged *ab initio* models were then filtered with DAMFILT.

### Molecular dynamics (MD) simulations

MD simulations for the Rtt109–Vps75_2_ subunit were carried out with GROMACS 5.1.1^63^, using the AMBER99SB-ILDN parameter set^64^ and periodic boundary conditions in all directions. The experimental structure of Rtt109–Vps75_2_ (PDB entry 3q66) was used as starting conformation. The complex was placed in a dodecahedral box, which was subsequently filled with explicit TIP3P water molecules and an appropriate number of counter-ions to neutralize the total charge. The system was minimized with a steepest-descent algorithm prior to two 1-ns steps of solvent equilibration at 298 K, first in a canonical ensemble and then in a NPT ensemble. Unrestrained MD simulations were then performed for a total of 400 ns. Temperature- and pressure-couplings were achieved by velocity rescaling and Parrinello-Rahman algorithms, respectively. Electrostatic interactions were treated with the particle mesh Ewald (PME) algorithm, while short-range non-bonded interactions were truncated at 0.9 nm, applying dispersion corrections to both energy and pressure to account for the truncation. 10 structures were selected as the ensemble of conformations representing the Rtt109–Vps75_2_ building block (Supplementary Fig. 5).

MD simulations for the Asf1–H3:H4–Rtt109–Vps75_2_ complexes were performed at 300 K using AMBER 2018^65^. The experimental structure of the complex, calculated as described above, was used as starting conformation. Prior to the MD runs, the structure was modified by the covalent addition of the H3^35-59^ tail with Chimera Modeller^66^ and by the non-covalent addition of acetyl coenzyme A (acCoA) with PYMOL^67^. During this second modification, the Rtt109 unit from the complex model was aligned and partially replaced with the Rtt109 structure from PDB entry 3q35. The replaced coordinates included residues 191–213, corresponding to the acCoA binding loop.

The system was simulated in explicit TIP3P water^68^, applying the AMBER14-SB force-field together with the general atom force-field. The system consisted of a cubic box of dimensions 138.22, 134.21 and 117.14 Å and contained a total of ~187000 atoms. The box encompassed a water-layer of 14-Å thickness to account for instabilities in the electrostatic-potential. Partial charges and specific improper angles of acCoA and acetylated lysine were generated according to Papamokos et al.^69^. The total charge of the system was neutralized by the addition of Na^+^ ions. Additional Na^+^ and Cl^–^ ions were also included to reach an overall NaCl concentration close to the experimental value of 150 mM.

Prior to the MD simulation all complexes were minimized with the following procedure. The water was minimized first (20,000 cycles) constraining the complex (NPT), and subsequently heated to 300 K (NVT). Next, the complete system was minimized (20,000 cycles), and heated to 300 K. Finally, the system was relaxed with restraints on the protein heavy atoms (NPT, 300 K, 0.5 ns). 10 ns equilibration MD were performed before the production runs.

This procedure was applied also to the final structural model from M3, before deposition as PDB entry 6o22.

The complex with the H3^35–59^ and acCoA modifications was subjected to an additional 100 ns of MD with center-of-mass restraints and a restraint between H3-K56 and acCoA, in order to place the H3-K56 in the Rtt109 active center.

Two modified complex structures (with the extended H3^35–59^ conformation or the H3^35–59^ conformation that allows H3-K56 to reach the Rtt109 active center) were then subjected to 100-ns MD simulation runs with center-of-mass restraints between the proteins only. These production runs were analyzed to study the conformational preference of the H3 N-terminal region and its dependence on the starting conformation (Fig. 3 and Supplementary Fig. 8).

### Acetylation assays

The Asf1−H3:H4 and Rtt109−Vps75_2_ sub-complexes were reconstituted by mixing the individual components followed by SEC (Superdex S200 10/300; 10 mM HEPES, pH 8.0, 0.1 M NaCl, 5 mM BME for Rtt109−Vps75_2_; 10 mM HEPES, pH 8.0, 0.5 M NaCl, 5 mM BME for Asf1−H3:H4). Acetylation reactions were performed using 0.2 μM Rtt109–Vps75_2_ sub-complexes, 0.2 μM Asf1−H3:H4 and 2 μM Ac-CoA in 10 mM HEPES pH 8.0, 100 mM NaCl. Reactions were stopped with 0.5 M sodium acetate, pH 5.5; 1.6 μL of the reaction mixtures were spotted on a nitrocellulose membrane. Membranes were air-dried for ca. 30 min, blocked for 30 min in 5% nonfat dry milk–TBST buffer (20 mM Tris–HCl pH 7.5, 150 mM NaCl, 0.05% Tween-20), and subsequently incubated for 1 h in a 1:2000 dilution of primary antibody in TBST: rabbit anti-histone H3 (acetyl K9) (ab4441 Abcam, lot no. GR3229436-1) or rabbit anti-histone H3 (acetyl K56) (SAB5600015 Sigma, lot no. P1100739). The membranes were then washed three times with TBST buffer and incubated for 1 h with the secondary antibody, anti-rabbit IgG (whole molecule) alkaline phosphatase (A3687 Sigma, lot no. SLBK3154V), 1:10,000 solution in TBST. After three washes with TBST buffer, the blots were developed with BCIP®/NBT Liquid Substrate System (B1911 Sigma, lot no. SLBS4876). The density of the dots was quantified using the ImageJ software^70^. The control reaction with 0.2 μM Asf1−H3:H4 did not give any signal above the background. An additional negative control with 6 μM Asf1−H3:H4, which corresponds to column “c” in the blots of Figs. 2, 3, and 5, was used for the normalization of the dots density and comparison of the experimental repeats. The results are the average of four experiments with the standard error of the mean representing the error.

### Reporting summary

Further information on research design is available in the Nature Research Reporting Summary linked to this article.

## Supplementary information

Supplementary Information

Peer Review

Reporting Summary

Source Data

## Data Availability

Coordinates of the Asf1–H3:H4–Rtt109–Vps75_2_ have been deposited to the Protein Data Bank under accession code 6o22. SANS data have been deposited to SASBDB under accession codes SASDFL3, SASDFM3, SASDFN3, SASDFP3, SASDFQ3, SASDFR3 [https://www.sasbdb.org/project/715/], SASDFK7, SASDFL7, SASDFM7, SASDFN7, SASDFP7, SASDFQ7 [https://www.sasbdb.org/project/822/]; chemical shift data have been deposited to BMRB under accession code 30576. The source data underlying Figs. 2e, 2f, 3c, 3d, 4b, 5b, 5c, 5d, 5e and Supplementary Figs. 1e, 7c, and 9b are provided as a Source Data file. All other relevant data are available from the authors.
