# Supplementary material for: Histone chaperone exploits intrinsic disorder to switch acetylation specificity
Source: Nat Commun. 2019 Aug 6;10:3435. doi: 10.1038/s41467-019-11410-7 (PMC6684614; doi:10.1038/s41467-019-11410-7)
Supplement: Supplementary file 1 — Supplementary Information [file 41467_2019_11410_MOESM1_ESM.pdf]

Supplementary Information

Histone chaperone exploits intrinsic disorder to switch acetylation specificity

Nataliya Danilenko[1], Lukas Lercher[1], John Kirkpatrick[1,2], Frank Gabel[3,4], Luca Codutti[1], Teresa Carlomagno[1,2*].

[1] Leibniz University Hannover, Centre for Biomolecular Drug Research, Schneiderberg 38, D-30167 Hannover, Germany.
[2] Helmholtz Centre for Infection Research, Group of Structural Chemistry, Inhoffenstrasse 7, D-38124 Braunschweig, Germany.
[3] University Grenoble Alpes, CEA, CNRS IBS, 71 avenue des Martyrs, F-38044 Grenoble, France.
[4] Institut Laue-Langevin, 71 avenue des Martyrs, F-38042 Grenoble, France.
* Correspondence: teresa.carlomagno@oci.uni-hannover.de

Supplementary Figures

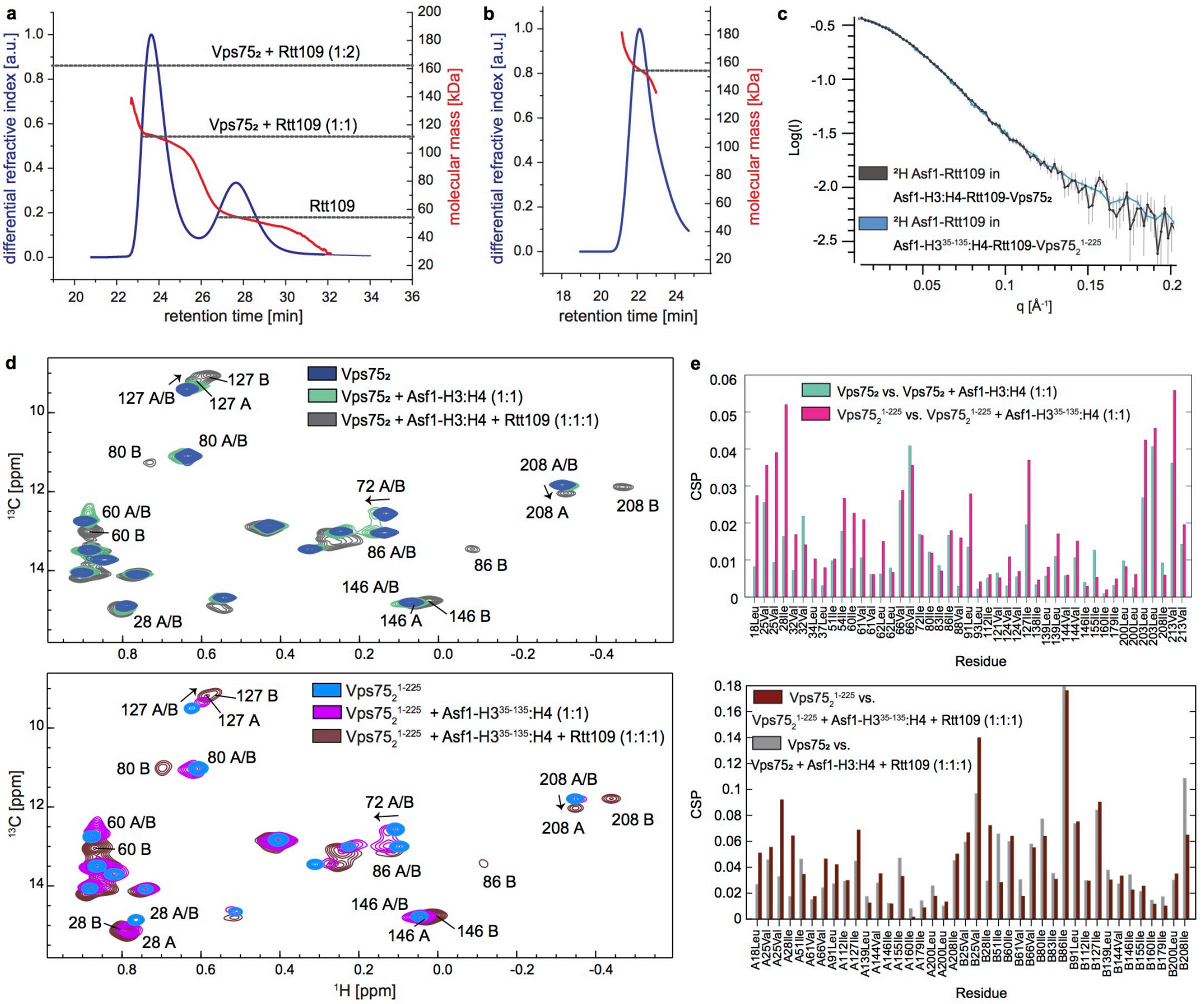

Supplementary Figure 1. Assembly of the *Sc* Asf1–H3:H4–Rtt109–Vps75$_2$ and Asf1–H3$^{35–135}$:H4–Rtt109–Vps75$_2$$^{1–225}$ complexes. (a) SEC-MALS analysis of the Rtt109:Vps75 1:1 mixture at 50 μM Rtt109. Red, molecular mass over the elution peaks; dashed grey lines, theoretical molecular masses of the corresponding complexes. Only one copy of Rtt109 binds the Vps75$_2$ homodimer. **(b)** SEC-MALS analysis of the *Sc* Asf1–H3:H4–Rtt109–Vps75$_2$ complex at 40 μM confirms the stoichiometry. **(c)** Comparison of SANS curves of the Asf1–H3$^{35–135}$:H4–Rtt109–Vps75$_2$$^{1–225}$ (blue) and Asf1–H3:H4–Rtt109–Vps75$_2$ (grey) complexes, assembled with ^{2}H-Rtt109/^{2}H-Asf1 in 42%:58% D_2O:H_2O buffer. The curves are scaled according to the concentration. **(d)** Overlay of ^{1}H-^{13}C HMQC spectra of Vps75$_2$ in isolation and upon subsequent addition of Asf1–H3:H4 and Rtt109 (top); overlay of ^{1}H-^{13}C HMQC spectra of Vps75$_2$$^{1–225}$ in isolation and upon subsequent addition of Asf1–H3$^{35–135}$:H4 and Rtt109 (bottom). The CSPs of the Vps75 structured domains follow the same pattern in both Asf1–H3:H4–Rtt109–Vps75$_2$ and Asf1–H3$^{35–135}$:H4–Rtt109–Vps75$_2$$^{1–225}$. **(e)** CSP analysis of the spectra in (d). Spectra were recorded on 60 μM Vps75$_2$ in 50 mM sodium citrate pH 6.5, 150 mM NaCl, 5 mM BME at 850 MHz and 298 K. The titration of Vps75$_2$ used a D_2O-based buffer, while that for Vps75$_2$$^{1–225}$ used an H_2O-based buffer. The small differences in the exact values of the CSPs between the complex assembled with Vps75$_2$ and that assembled with Vps75$_2$$^{1–225}$ are due to the isotopic effects induced by the different solvents. Source data are provided as a Source Data file.

```
a
H3 Xen  MARTKQTARKSTGGKAPRKQLATKAARKSAPATGGVKKPHRYRPGTVALR 50
H3 Xen# MARTKQTARKSTGGKAPRKQLATKAARKSAPATGGVKKPHRYRPGTVALR 50
H3 Sc   MARTKQTARKSTGGKAPRKQLASKAARKSAPSTGGVKKPHRYKPGTVALR 50
        **********************:********:**********:*******

H3 Xen  EIRRYQKSTELLIRKLPFQRLVREIAQDFKTDLRFQSSAVMALQEASEAY 100
H3 Xen# EIRRYQKSTELLIRKLPFQRLVREIAQDFKTDLRFQSSAVMALQEASEAY 100
H3 Sc   EIRRFQKSTELLIRKLPFQRLVREIAQDFKTDLRFQSSAIGALQESVEAY 100
        ****:**********************************: ****: ***

H3 Xen  LVGLFEDTNLCAIHAKRVTIMPKDIQLARRIRGERA 136
H3 Xen# LVALFEDTNLCAIHAKRVTIMPKDIQLARRIRGERA 136
H3 Sc   LVSLFEDTNLAAIHAKRVTIQKKDIKLARRLRGERS 136
        **.*******.*********  ***:****:****:

H4 Xen  MSGRGKGGKGLGKGGAKRHRKVLRDNIQGITKPAIRRLARRGGVKRISGL 50
H4 Xen# MSGRGKGGKGLGKGGAKRHRKVLRDNIQGITKPAIRRLARRGGVKRISGL 50
H4 Sc   MSGRGKGGKGLGKGGAKRHRKILRDNIQGITKPAIRRLARRGGVKRISGL 50
        *********************:****************************

H4 Xen  IYEETRGVLKVFLENVIRDAVTYTEHAKRKTVTAMDVVYALKRQGRTLYG 100
H4 Xen# IYEETRGVLKVFLENVIRDAVTYTEHAKRKTVTAMDVVYALKRQGRTLYG 100
H4 Sc   IYEEVRAVLKSFLESVIRDSVTYTEHAKRKTVTSLDVVYALKRQGRTLYG 100
        ****.*.*** ***.****:*************::***************

H4 Xen  FGG 103
H4 Xen# FGG 103
H4 Sc   FGG 103
        ***
```

```
b
Sc  AQRDL-EDEE-GESGLSADGDSE-DDDGSLGEVDLPLSDEE----PSS--  259
Sp  AMTEEASDED---ESVDLEEDEE-EEDEE--DEEG---DEEKQE-PPS--  237
Ca  ALSP-DEDDD----ANDEEDTTEGEE------LDLSDDDVEE---PELPK  258
Cp  ALSNESGEED----SEEEEDTSEGEE------LDLSDVEVEG-------K  265
Cg  AQRDL-EDEEE-DDSE--DNSSE-E----PLDLEVESESEESD-------  256
Kl  AQRDVMDESD-GD-S---EDDSDA-----PLDLDLEEVNDEEGSDSKHLN  270

Sc  --------------KKRKV     264
Sp  --------------KKSKKSN   244
Ca  KRTLEEEENEHAT-KKSK      275
Cp  KRTLGDDIVDAPCEKKVKQSSSV 288
Cg  --------------KKRRKI    262
Kl  --------------KKTKLQ    276
```

Supplementary Figure 2. (a) Sequence alignment of the histone sequences of *Xenopus laevis* (Xen), those used in this study (Xen #) and *Saccharomyces cerevisiae* (Sc). **(b)** Manual sequence alignment of the CTADs of Vps75 orthologs from organisms whose histone acetylation is dependent on Rtt109: *Saccharomyces cerevisiae* (Sc), *Schizosaccharomyces pombe* (Sp), *Candida albicans* (Ca), *Candida parapsilosis* (Cp), *Candida glabrata* (Cg), *Kluyveromyces lactis* (Kl).

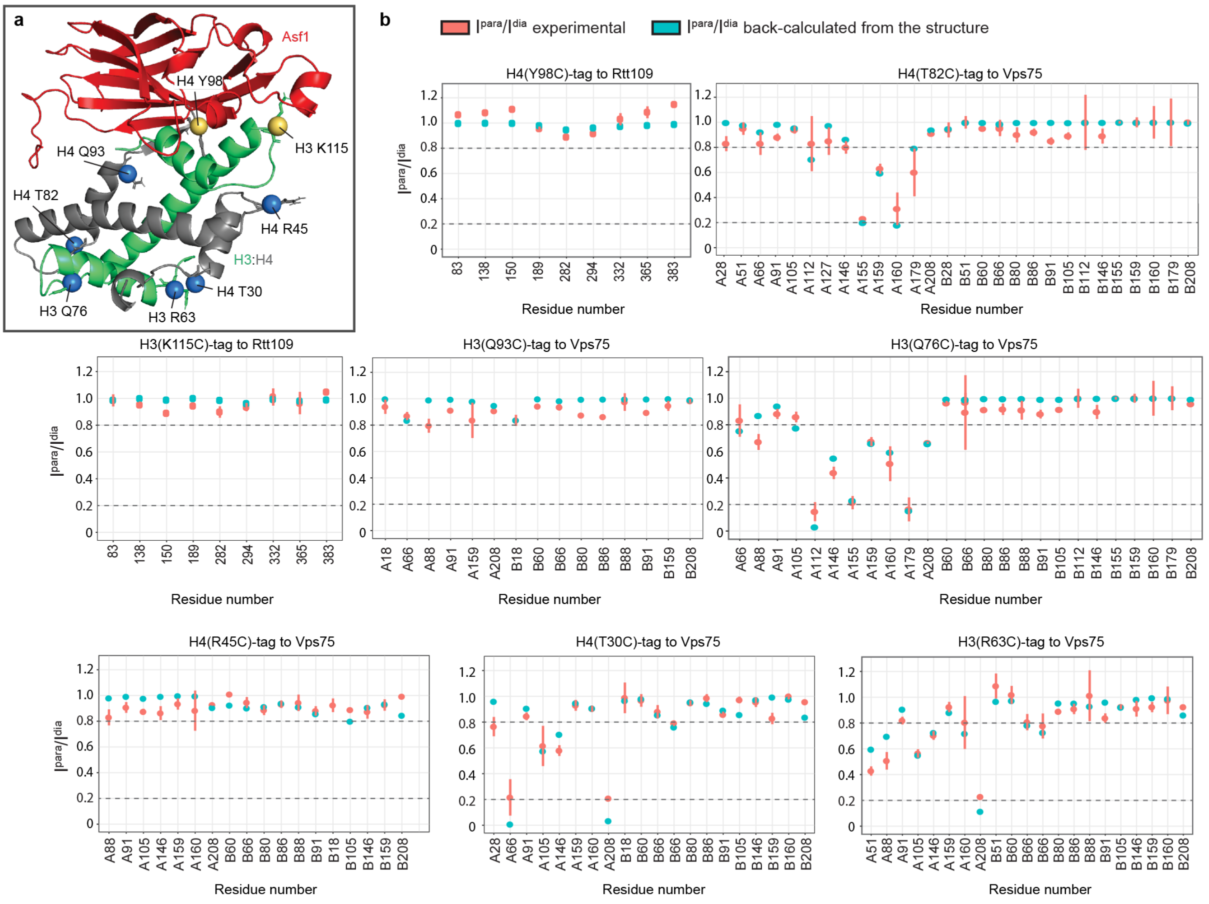

Supplementary Figure 3. Paramagnetic relaxation enhancements (PREs) (a) The positions of the paramagnetic tags are shown on the Asf1–H3:H4 subunit (PDB entry 2hue). Blue and yellow spheres indicate the tags used in PRE measurements of the ^{1}H-^{13}C methyl groups of Vps75 and Rtt109, respectively. **(b)** Comparison of experimental (red) and back-calculated (light blue) ratios of peak intensities measured in the presence of the paramagnetic (I^{para}) or diamagnetic (I^{dia}) forms of the tags.

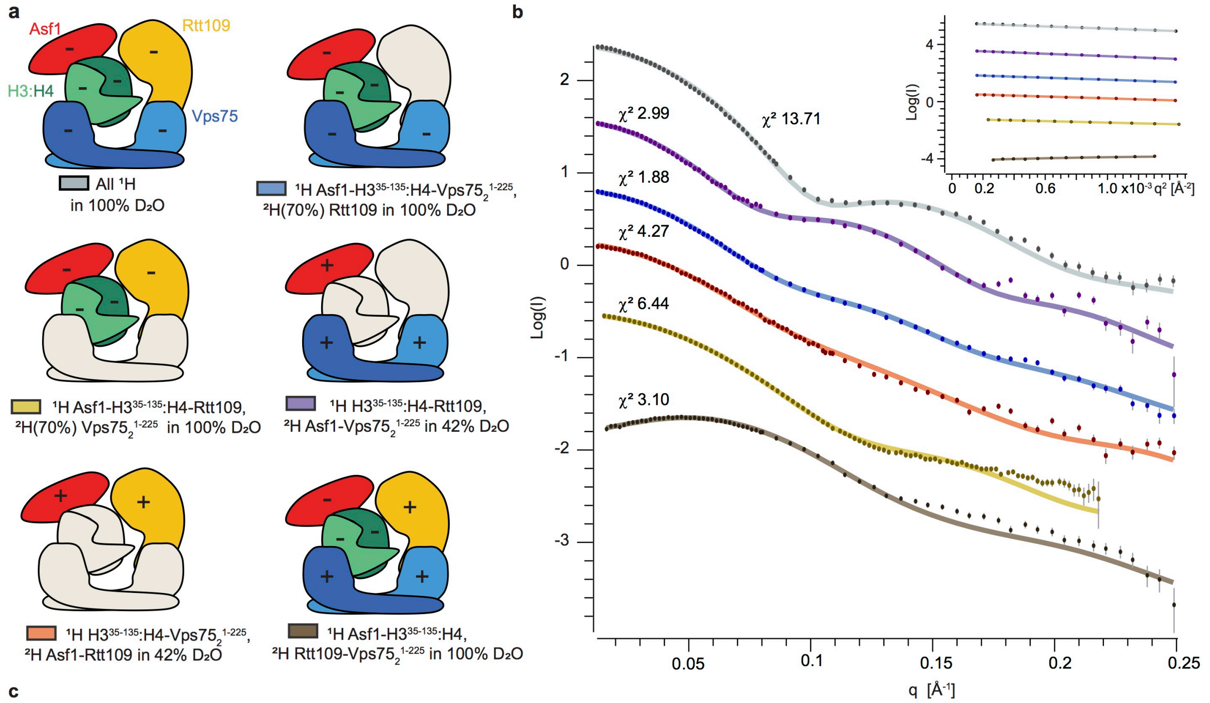

c

#	Sample	D_2O:H_2O [%]	Concentration [mg/mL]	R_g Guinier [Å]	R_g GNOM [Å]	D_{MAX} GNOM [Å]
1.	^{1}H Asf1-H3$^{35\text{-}135}$:H4-Rtt109-Vps75$_2^{1\text{-}225}$	100:0	2.35	35.26 ± 0.38	35.32	118
2.	^{1}H Asf1-H3$^{35\text{-}135}$:H4, ^{2}H Rtt109-Vps75$_2^{1\text{-}225}$	100:0	3.85	n.d.	n.d.	n.d.
3.	^{1}H Asf1-H3$^{35\text{-}135}$:H4-Vps75$_2^{1\text{-}225}$, ^{2}H(70%) Rtt109	100:0	3.8	32.82 ± 0.46	32.86	105
4.	^{1}H Rtt109-H3$^{35\text{-}135}$:H4, ^{2}H Asf1-Vps75$_2^{1\text{-}225}$	42:58	4.7	34.99 ± 0.46	36.02	110
5.	^{1}H Vps75$_2^{1\text{-}225}$-H3$^{35\text{-}135}$:H4, ^{2}H Asf1-Rtt109	42:58	5.2	30.60 ± 0.76	31.03	105
6.	^{1}H Asf1-H3$^{35\text{-}135}$:H4-Rtt109, ^{2}H(70%) Vps75$_2^{1\text{-}225}$	100:0	4.9	28.35 ± 0.18	28.40	95
7.	^{1}H Asf1-H3$^{35\text{-}135}$:H4, ^{2}H(70%) Rtt109-Vps75$_2^{1\text{-}225}$	100:0	2.55	19.43 ± 0.36	20.65	55
8.	^{1}H Asf1-H3:H4, ^{2}H Rtt109-Vps75$_2^{1\text{-}225}$	42:58	4.0	33.65 ± 0.28	33.24	105
9.	^{1}H Asf1-H3:H4-Rtt109, ^{2}H Vps75$_2^{1\text{-}225}$	42:58	4.9	27.41 ± 0.32	27.81	90
10.	^{1}H Vps75$_2$-H3:H4, ^{2}H Asf1-Rtt109	42:58	3.25	30.82 ± 0.34	30.88	95
11.	^{1}H Asf1-H3:H4-Rtt109-Vps75$_2$	100:0	2.73	34.96 ± 0.40	34.53	115
12.	^{1}H Asf1-H3:H4-Rtt109-Vps75$_2^{1\text{-}225}$	100:0	1.92	35.01 ± 0.35	34.31	110

Supplementary Figure 4. Experimental SANS curves and their fits to those predicted from the Asf1–H3$^{35–135}$:H4–Rtt109–Vps75$_2^{1–225}$ complex structure. (a) Schematic representation of the SANS complexes used in this study. The colored proteins contribute to the scattering-intensity with either positive (+) or negative (–) contrast, while the grey proteins are effectively invisible due to contrast-matching with the solvent. **(b)** Fits of the experimental curves with those predicted from the structure of Fig. 1. **(c)** Summary of the SANS measurements and the resulting structural parameters. Experiments 1–6 (grey) were used to select the final structures from M3 (Supplementary Fig. 6); experiments 7–9 (cyan) were used to select the structures of the Asf1–H3:H4 and Rtt109–Vps75$_2$ sub-complexes prior to M3 (Supplementary Fig. 5); experiment 10 (white) was not used in the structure calculations and was used for cross-validation; experiments 1, 11 and 12 were used to calculate the *ab initio* envelopes of Fig. 5.

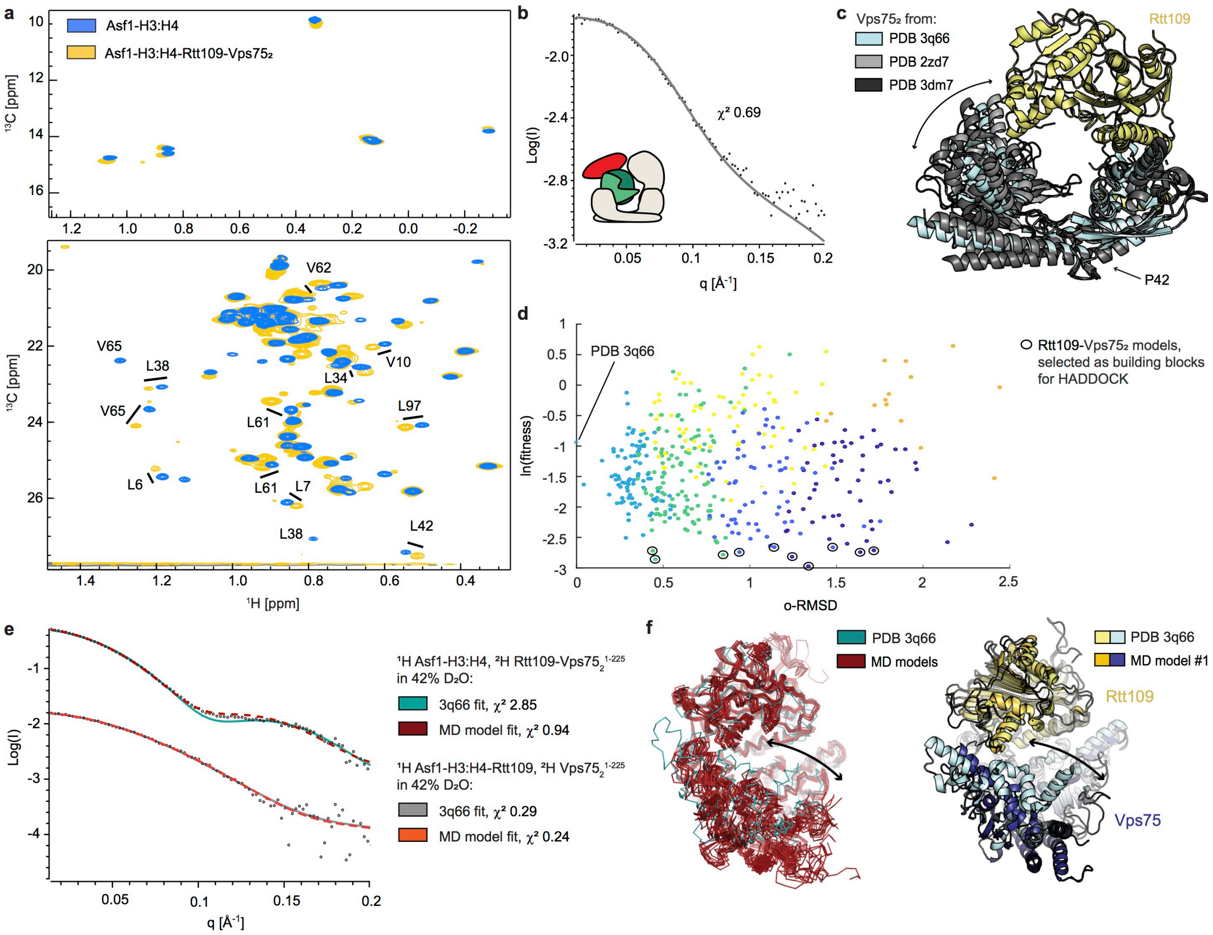

Supplementary Figure 5. (a, b) The Asf1–H3:H4 structure is conserved in complex with Rtt109–Vps75$_2$. (a) Overlay of ^{1}H-^{13}C HMQC spectra of ILV methyl-labeled Asf1 as part of Asf1–H3:H4 and Asf1–H3:H4–Rtt109-Vps75$_2$ (top: Ile region; bottom: Leu/Val region). CS differences between the two spectra are observed only for the Asf1 surface containing amino acids ~35–75, which was previously shown to bind the Rtt109 C-terminal tail[37]. Spectra were recorded on 50 μM Asf1 in 50 mM sodium citrate pH 6.5, 150 mM NaCl, 5 mM BME at 850 MHz and 298 K. **(b)** Overlay of the experimental SANS curve acquired for Asf1–H3$^{35–135}$:H4–Rtt109–Vps75$_2$ in 100% D_2O buffer, using ^{1}H-Asf1–H3$^{35–135}$:H4 and ^{2}H(70%)-Rtt109–Vps75, with the curve predicted from the structure of the Asf1–H3:H4–Rtt109–Vps75$_2$ complex generated using PDB entry 2hue as the Asf1–H3:H4 building block for M3. **(c–f). Selection of the Rtt109–Vps75$_2$ structures as building blocks for M3. (c)** Overlay of three crystal structures containing Vps75$_2$, demonstrating the variability of the hinge angle at residue P42. **(d)** Plot of the normalized χ^2 (see Methods for definition) versus o-RMSD (orientational root-mean-square deviation) to the structure of PDB entry 3q66 for the MD-generated conformers of the Rtt109–Vps75$_2$ complex. The o-RMSD was calculated for Vps75(A) relative to Rtt109. The normalized χ^2 was calculated using the SANS curves of Asf1–H3:H4–Rtt109–Vps75$_2^{1–225}$, acquired with ^{2}H-Vps75$^{1–225}$ or ^{2}H-Rtt109 and ^{2}H-Vps75$^{1–225}$ in 42%:58% D_2O:H_2O buffer. The conformers are clustered by o-RMSD and the clusters are shown in different colors. The ten structures with lowest normalized χ^2, which contain representatives from the three best-scoring clusters, were selected as building blocks for M3. **(e)** Fits to the experimental SANS curves of the MD model with lowest normalized χ^2 and of the structure from PDB entry 3q66. **(f)** Structural variability of the ten selected conformers. The right panel displays the structural differences between the best MD model and PDB entry 3q66, which result in a more open cavity for substrate binding. The left panel shows that all selected models adopt the more open conformation.

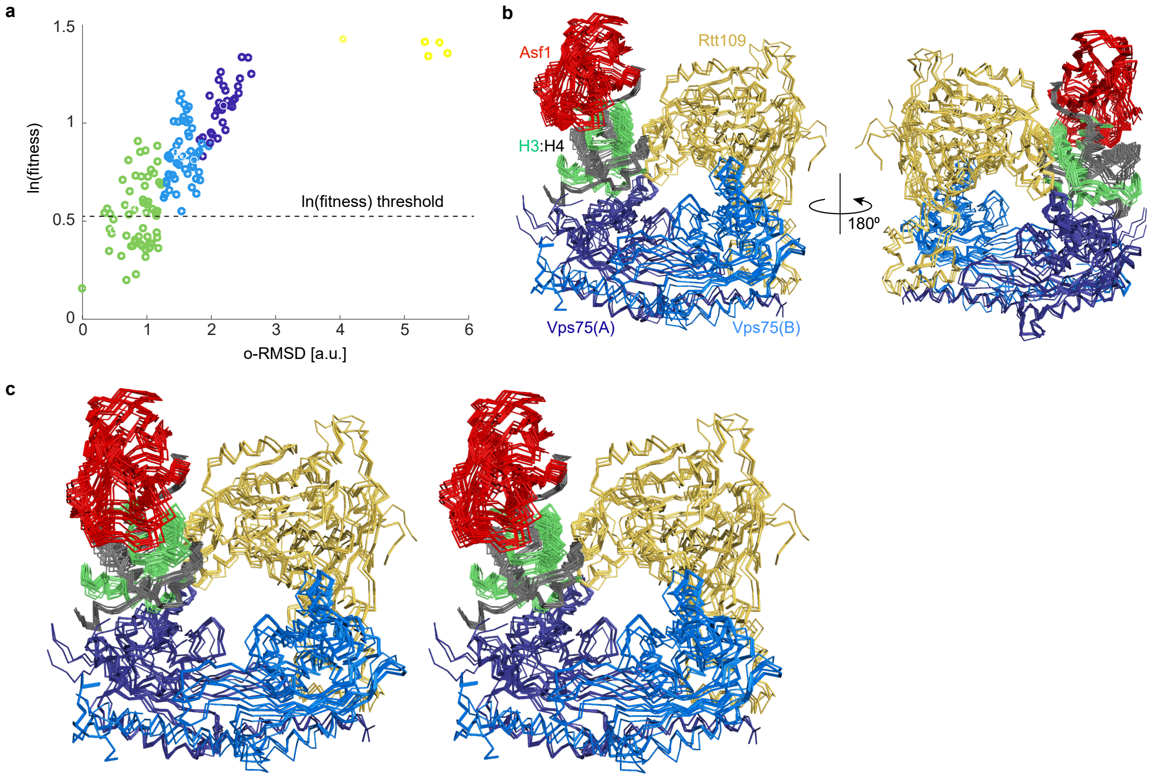

Supplementary Figure 6. Selection of the final ensemble. (a) Correlation between the structural similarity (orientational root-mean-square deviation, o-RMSD) of the conformations calculated by the M3 protocol and their agreement to the experimental SANS data, measured by the fitness parameter (Methods). The o-RMSD was calculated with respect to the structure with the best fitness; the structures were clustered by o-RMSD and each cluster is represented by one color. Members of the cluster that best fit the experimental data (green cluster) below the indicated threshold were selected as the final ensemble (33 structures). **(b)** Superposition of the ten best-fitness structures of the final ensemble. **(c)** Stereo view of the superposition of the ten best-fitness structures of the final ensemble.

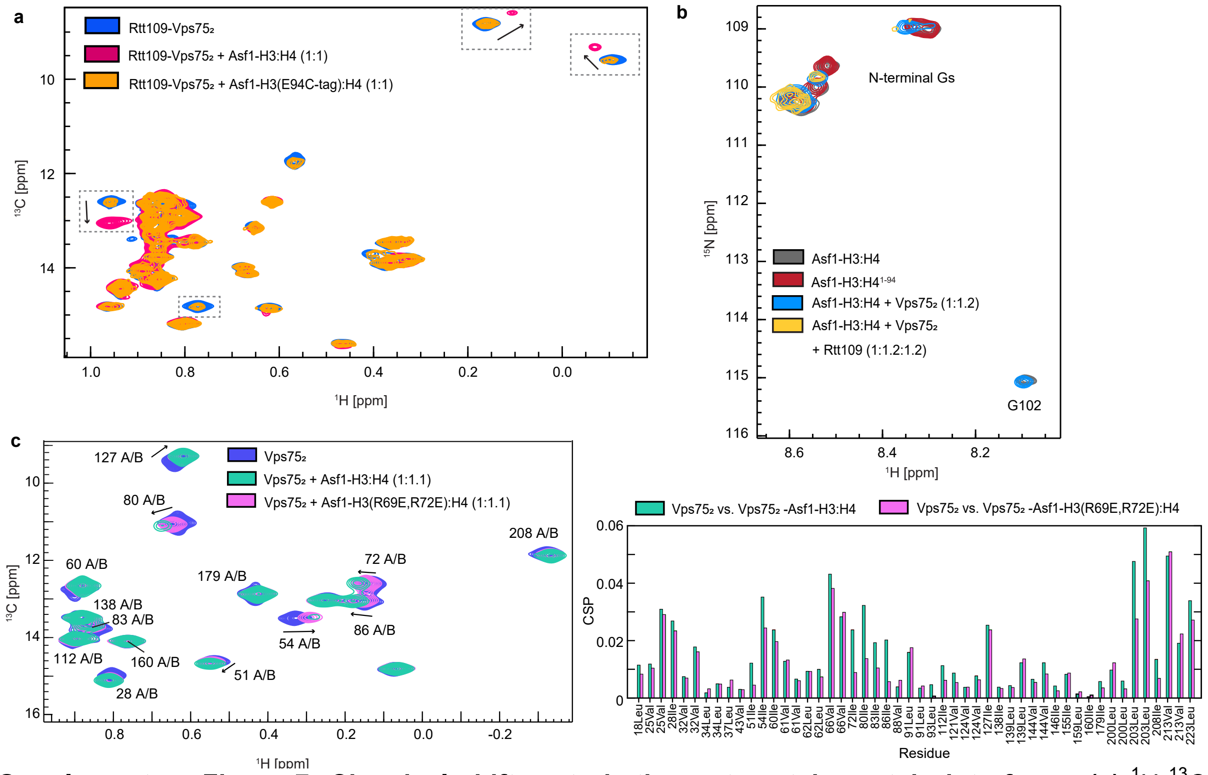

Supplementary Figure 7. Chemical shift perturbations at protein-protein interfaces. (a) ^{1}H-^{13}C HMQC spectra of I-labeled Rtt109 as part of the Rtt109–Vps75$_2$ complex upon titration of Asf1–H3:H4 (pink) or Asf1–H3(E94C-tag):H4 (orange). "E94C-tag" denotes the presence of a paramagnetic tag coupled with the cysteine residue engineered at position 94 of H3. The peaks in the boxes display noticeable CSPs upon addition of wild-type Asf1–H3:H4, but not Asf1–H3(E94C-tag):H4. The spectra were recorded on ~65 μM Rtt109 in 50 mM sodium citrate pH 6.5, 150 mM NaCl, 5 mM BME at 850 MHz and 298 K. **(b)** ^{1}H-^{15}N HSQC of ^{15}N-labeled H4 in the Asf1–H3:H4 complex upon titration of Vps75 (blue) and Rtt109 (yellow). Missing peaks of ^{15}N-labeled H4$^{1–94}$ in Asf1–H3:H4$^{1–94}$ (red) belong to the C-terminal region of H4 (H4$^{95–102}$). The spectra were recorded on 50 μM Asf1–H3:H4 in 50 mM sodium citrate pH 6.5, 150 mM NaCl, 5 mM BME at 600 MHz and 298 K. (**c**) ^{1}H-^{13}C HMQC spectrum (left) and CSP plot (right) of ILV-labeled Vps75$_2$ in the presence of Asf1–H3:H4 or Asf1–H3(R69E,R72E):H4. The spectra were recorded on 60 μM Vps75 in 50 mM sodium citrate pH 6.5, 150 mM NaCl, 5 mM BME at 850 MHz and 298 K. Source data are provided as a Source Data file.

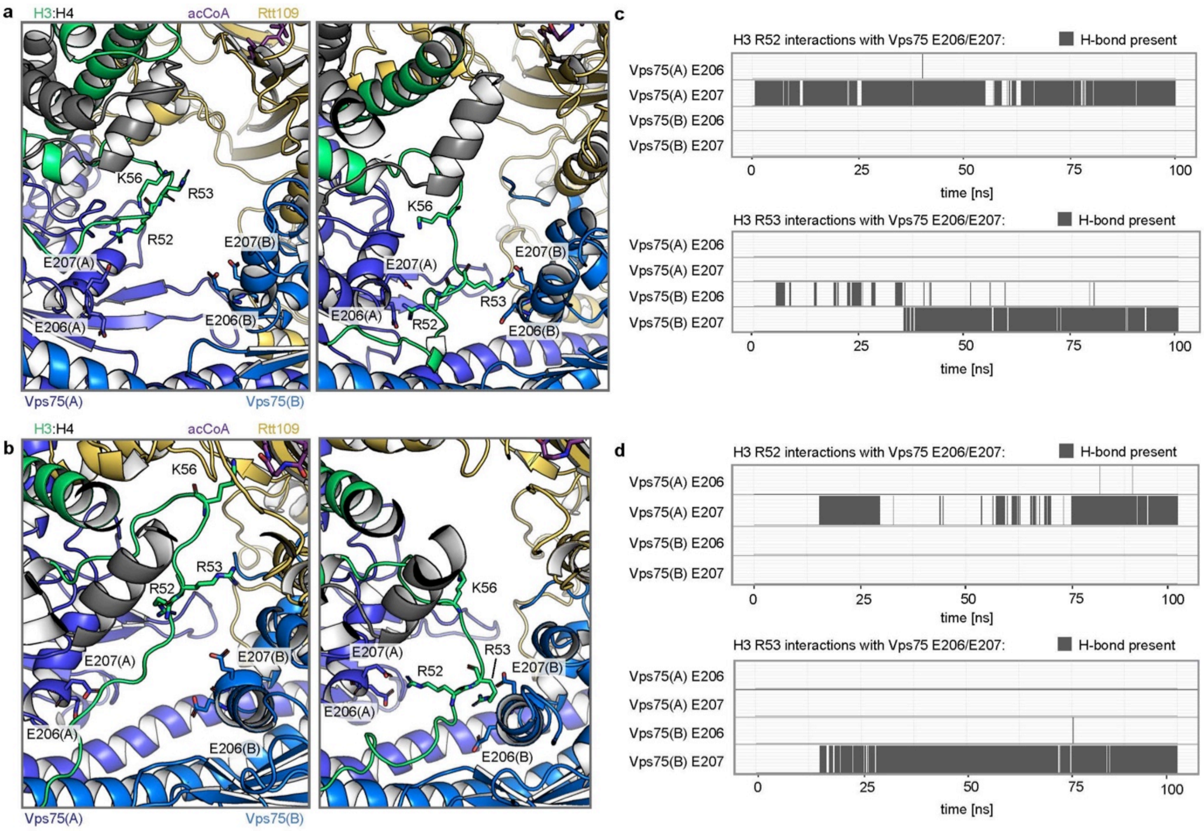

Supplementary Fig. 8. The acidic patch of Vps75 keeps K56 away from the Rtt109 catalytic pocket. (a) Right, final conformation of the MD simulation trajectory of Asf1–H3$^{35–135}$:H4–Rtt109–Vps75$_2^{1–225}$, starting from the structure (left) of Fig. 1 with the stretch H3$^{35–59}$ built in an extended conformation, as in Fig. 3. **(b)** Right, final conformation of the MD simulation trajectory of Asf1–H3$^{35–135}$:H4–Rtt109–Vps75$_2^{1–225}$, starting from the structure (left) of Fig. 1 with the stretch H3$^{35–59}$ modeled by placing K56 in the catalytic pocket of Rtt109. Both MD simulations in (a) and (b) resulted in the formation of stable H-bonds between H3-R52 and Vps75(A)-E207 as well as between H3-R53 and Vps75(B)-E207, which are incompatible with H3-K56 reaching the Rtt109 catalytic center. **(c)** Analysis of H-bonds present between H3-R52 or R53 and Vps75-E206 or E207 in the 100-ns MD simulations of Asf1–H3$^{35–135}$:H4–Rtt109–Vps75$_2^{1–225}$ as in (a) and Fig. 3. **(d)** Analysis of H-bonds present between H3-R52 or R53 and Vps75-E206 or E207 in the 100-ns MD simulations of Asf1–H3$^{35–135}$:H4–Rtt109–Vps75$_2^{1–225}$ as in (b).

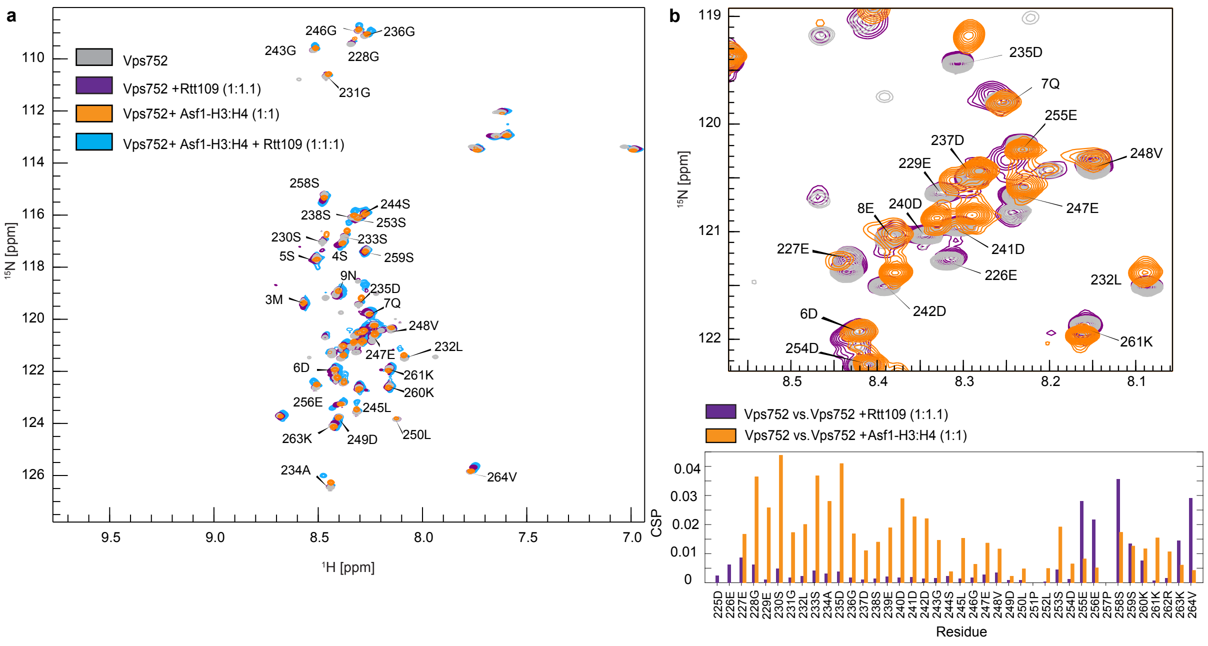

Supplementary Fig. 9. (a) Overlay of ^{1}H-^{15}N HSQC spectra of ^{15}N-labeled Vps75$_2$ in isolation (grey), in complex with Rtt109 (purple) and as part of either the Asf1–H3:H4–Vps75$_2$ (orange) or the Asf1–H3:H4–Rtt109–Vps75$_2$ (blue) complex. The chemical shift dispersion of the Vps75$^{226–264}$ stretch remains very narrow indicating that the Vps75 CTAD stays disordered in all complexes. **(b)** Top, expansion of the overlay of ^{1}H-^{15}N HSQC spectra of Vps75$_2$ in isolation (grey), in complex with Rtt109 (purple) and as part of Asf1–H3:H4–Vps75$_2$ (orange). Bottom, the CSP analysis demonstrates that the interaction between the Vps75$_2$ CTADs and Rtt109 affects different amino acids from those affected by the interaction with Asf1–H3:H4. Spectra were recorded on 50 μM Vps75 in 50 mM sodium citrate pH 5.5, 150 mM NaCl, 5 mM BME at 600 MHz and 298 K. Source data are provided as a Source Data file.

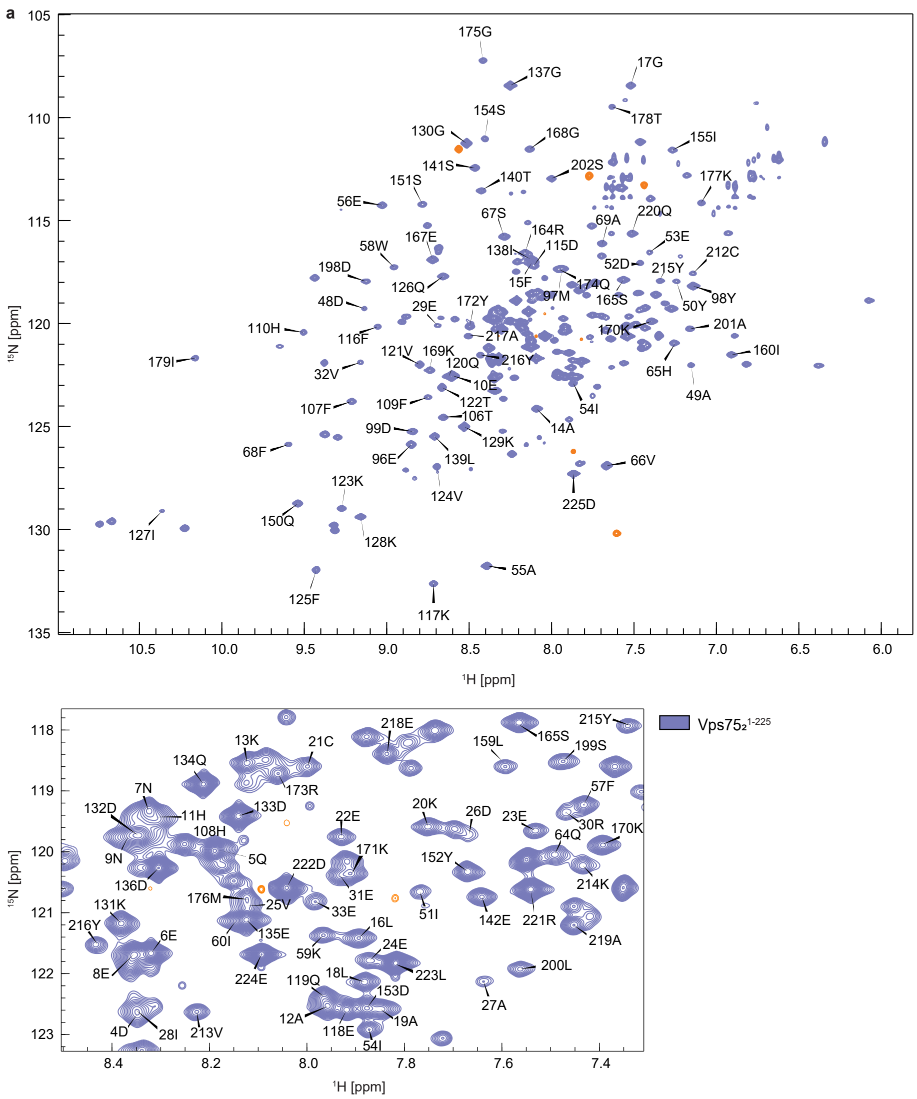

Supplementary Figure 10. Backbone assignment of free Vps75$_2^{1\text{-}225}$. ^{1}H-^{15}N HSQC spectrum of 0.8 mM free Vps75$_2^{1\text{-}225}$ (0.8 mM) in 20 mM Tris–HCl pH 7.5, 200 mM NaCl, 1 mM DTT at 850 MHz and 300 K. The upper panel shows the full spectrum; the lower panel an excerpt thereof.

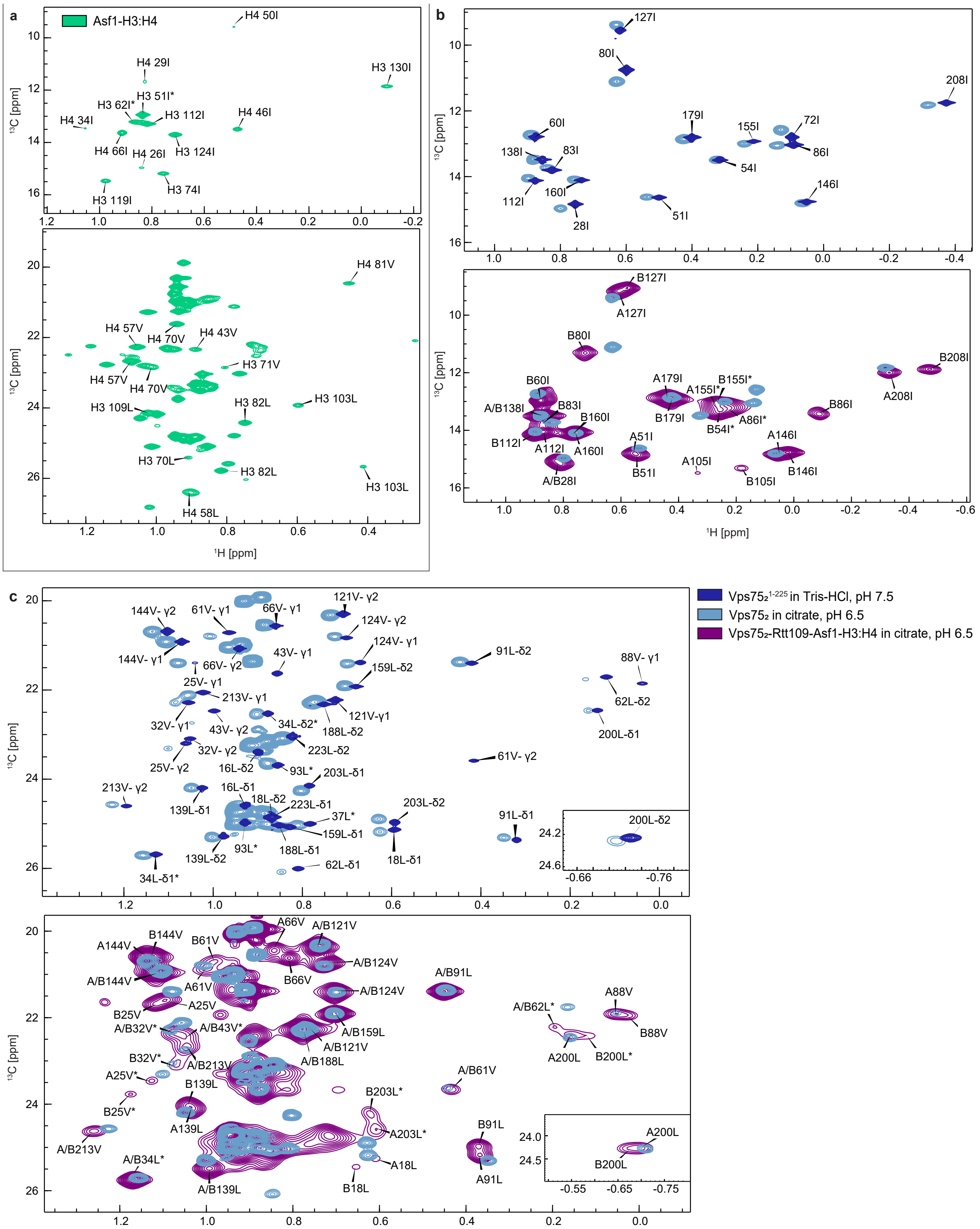

Supplementary Figure 11. Assignment of the methyl resonances of H3:H4 and Vps75. (a) ^{1}H-^{13}C HMQC spectrum of ILV-labelled H3:H4 as part of the Asf1–H3:H4 complex (0.66 mM) in 20 mM Tris–HCl pH 7.5, 1 M NaCl, 1 mM Na-EDTA, 5 mM BME in 100% D2O at 850 MHz and 298 K. **(b, c)** ^{1}H-^{13}C HMQC spectra of ILV-labelled Vps75$_2$ (light blue) and Vps75$_2$ as part of the Asf1–H3:H4–Rtt109–Vps75$_2$ complex (purple) in 50 mM sodium citrate pH 6.5, 150 mM NaCl, 5 mM BME at 850 MHz and 298 K (**b**: Ile region, **c**: Leu/Val region with stereospecific assignment). The assignments are shown for the Vps75$_2$ and the Vps75$_2$ in the Asf1–H3:H4–Rtt109–Vps75$_2$ complex. In the latter spectrum, A and B indicate the Vps75(A) and Vps75(B) chains, respectively. The asterisk marks tentative assignments.
