# Supplementary material for: Histone chaperone exploits intrinsic disorder to switch acetylation specificity
Source: Nat Commun. 2019 Aug 6;10:3435. doi: 10.1038/s41467-019-11410-7 (PMC6684614; doi:10.1038/s41467-019-11410-7)
Supplement: Supplementary file 3 — Reporting Summary [file 41467_2019_11410_MOESM3_ESM.pdf]

nature research

Corresponding author(s): Teresa Carlomagno

Last updated by author(s): Jul 10, 2019

Reporting Summary

Nature Research wishes to improve the reproducibility of the work that we publish. This form provides structure for consistency and transparency in reporting. For further information on Nature Research policies, see Authors & Referees and the Editorial Policy Checklist.

Statistics

For all statistical analyses, confirm that the following items are present in the figure legend, table legend, main text, or Methods section.

n/a	Confirmed	
☐	☒	The exact sample size (*n*) for each experimental group/condition, given as a discrete number and unit of measurement
☐	☒	A statement on whether measurements were taken from distinct samples or whether the same sample was measured repeatedly
☒	☐	The statistical test(s) used AND whether they are one- or two-sided<br>*Only common tests should be described solely by name; describe more complex techniques in the Methods section.*
☒	☐	A description of all covariates tested
☐	☒	A description of any assumptions or corrections, such as tests of normality and adjustment for multiple comparisons
☐	☒	A full description of the statistical parameters including central tendency (e.g. means) or other basic estimates (e.g. regression coefficient) AND variation (e.g. standard deviation) or associated estimates of uncertainty (e.g. confidence intervals)
☒	☐	For null hypothesis testing, the test statistic (e.g. *F*, *t*, *r*) with confidence intervals, effect sizes, degrees of freedom and *P* value noted<br>*Give P values as exact values whenever suitable.*
☒	☐	For Bayesian analysis, information on the choice of priors and Markov chain Monte Carlo settings
☒	☐	For hierarchical and complex designs, identification of the appropriate level for tests and full reporting of outcomes
☒	☐	Estimates of effect sizes (e.g. Cohen's *d*, Pearson's *r*), indicating how they were calculated

Our web collection on statistics for biologists contains articles on many of the points above.

Software and code

Policy information about availability of computer code

Data collection	Topspin 3.2
Data analysis	NMR data analysis: NMRPipe 8.7, Topspin 3.2, CcpNmr Analysis v2.4, FuDA; Structure calculation: CNS 1.3, HADDOCK 2.2, M3 protocol available at https://github.com/ezgikaraca/ISD-files; SANS data: ATSAS 2.7.1 (PRIMUS, CRYSON, DAMMIF, DAMAVER, DAMFILT); MD: GROMACS 5.1.1, AMBER 2018; Chimera Modeller, PYMOL 2.1; Dot-blot analysis: ImageJ 1.50i;

For manuscripts utilizing custom algorithms or software that are central to the research but not yet described in published literature, software must be made available to editors/reviewers. We strongly encourage code deposition in a community repository (e.g. GitHub). See the Nature Research guidelines for submitting code & software for further information.

Data

Policy information about availability of data

All manuscripts must include a data availability statement. This statement should provide the following information, where applicable:

- Accession codes, unique identifiers, or web links for publicly available datasets
- A list of figures that have associated raw data
- A description of any restrictions on data availability

Coordinates of the Asf1–H3:H4–Rtt109–Vps752 have been deposited to the Protein Data Bank under accession code 6O22. SANS data have been deposited to SASBDB under accession codes SASDFL3, SASDFM3, SASDFN3, SASDFP3, SASDFQ3, SASDFR3, SASDFK7, SASDFL7, SASDFM7, SASDFN7, SASDFP7, SASDFQ7; chemical shift data have been deposited to BMRB under accession code 30576. The source data underlying Figs 2e, 2f, 3c, 3d, 4b, 5c, 5d, 5e and Supplementary Figs 1e, 7c and 9b are provided as a Source Data file. All other relevant data are available from the authors. Associated raw data not provided in the Source Data file: 1) FID of NMR spectra: Figure 1, Figure 4, Figure 5, Supplementary Figs. 1, 5, 7, 9, 10, 11. These raw data are available from the authors upon request.

Field-specific reporting

Please select the one below that is the best fit for your research. If you are not sure, read the appropriate sections before making your selection.

☒ Life sciences ☐ Behavioural & social sciences ☐ Ecological, evolutionary & environmental sciences

For a reference copy of the document with all sections, see nature.com/documents/nr-reporting-summary-flat.pdf

Life sciences study design

All studies must disclose on these points even when the disclosure is negative.

Sample size	The sample size for each experiment is indicated in the figure legends.
Data exclusions	The first 3 data points of blots #3 and#4, corresponding to the 0.16 min time points, were excluded from the analysis shown in Fig. 3c/5d and Fig. 3d/5e .<br>These points showed elevated intensities (higher than those at later time points) in the blots stained with the K56-antibodies (Fig. 3c/5d); we attributed this to a pipetting error. Both blots #3 and #4 were done on the same day, in parallel. The same reaction mixtures were used for performing blots #3 and #4 stained with K9-antibodies for Fig. 3d/5e. Despite the fact that the 0.16 min time points of the K9-stained blots did not show elevated intensities, we eliminated them from the analysis, to avoid any bias in the comparison between K9ac and K56ac. Inclusion of these time points in the analysis increased the errors but did not change the conclusions.
Replication	Dot-blots were performed in quadruplicate, replication was consistent.
Randomization	n/a
Blinding	n/a

Reporting for specific materials, systems and methods

We require information from authors about some types of materials, experimental systems and methods used in many studies. Here, indicate whether each material, system or method listed is relevant to your study. If you are not sure if a list item applies to your research, read the appropriate section before selecting a response.

Materials & experimental systems

n/a	Involved in the study	
☐	☒	Antibodies
☒	☐	Eukaryotic cell lines
☒	☐	Palaeontology
☒	☐	Animals and other organisms
☒	☐	Human research participants
☒	☐	Clinical data

Methods

n/a	Involved in the study	
☒	☐	ChIP-seq
☒	☐	Flow cytometry
☒	☐	MRI-based neuroimaging

Antibodies

Antibodies used	rabbit anti-histone H3 (acetyl K9) ab4441 Abcam, lot no. GR3229436-1 (1:2000); rabbit anti-histone H3 (acetyl K56) SAB5600015 Sigma, lot no. P1100739 (1:2000);
Validation	Both antibodies were verified using a control reaction with acCoA and the substrate Asf1-H3:H4 in the absence of the enzyme Rtt109; rabbit anti-histone H3 (acetyl K56) was additionally validated using the H3 K56C mutant;<br>Validation by the manufacturer: ab4441 https://www.abcam.com/histone-h3-acetyl-k9-antibody-chip-grade-ab4441.html, http://compbio.med.harvard.edu/antibodies/antibodies/256, http://compbio.med.harvard.edu/antibodies/antibodies/105;
