# Supplementary material for: Histone chaperone exploits intrinsic disorder to switch acetylation specificity
Source: Nat Commun. 2019 Aug 6;10:3435. doi: 10.1038/s41467-019-11410-7 (PMC6684614; doi:10.1038/s41467-019-11410-7)
Supplement: Supplementary file 2 — Peer Review [file 41467_2019_11410_MOESM2_ESM.pdf]

Reviewers' comments:

Reviewer #1 (Remarks to the Author):

This manuscript presents an model of the yeast Asf1–H3:H4–Rtt109–Vps75 complex (160KDa) calculated by performing data-driven docking combined with MD simulations of the known structures of it's sub-complexes using SANS data and NMR-derived distance restraints. The ordered parts of the complex form a doughnut-like shape with a central cavity of ~25 Å width and the active site of the acetyltransferase Rtt109 positioned inside the cavity.
Rtt109 acetylates multiple substrates including K9, K14, K23, K27, and K56 of histone H3.

The authors focus on acetylation of K56, located just before the structured H3 core, and K9 located within the intrinsically disordered region (35aa) of H3. The authors have demonstrated that C-terminal acidic domain (CTAD, 40aa) of Vsp75 guides substrate selective interactions with the N-terminal tail of H3. Both, the CTAD of Vps75 and NTD of H3, are disordered and have opposite net charges (-11 vs +10). The authors speculate that non-specific, and predominantly electrostatic interactions between these two disordered domains results in the H3 tail being confined in the central cavity, thereby locating K9 in the proximity of the Rtt109 active site.

This work is very interesting and provides a detailed example of the growing appreciation of the fuzzy interactions that can regulate specific biological interactions.

The following points should be addressed, however, before publication.

1. The authors performed MD simulations of the truncated complex Asf1–H3(35-135):H4–Rtt109–Vps75(1-225) that is missing both CTAD of Vps75 and NTD of H3 domains. These simulations reveal interaction between H3 and Vps75 that keeps K56 away from the Rtt109 catalytic pocket. However even more interesting would be simulation of the CTAD of Vps75 and NTD of H3 domains which could potentially shed some light on the "fuzzy interactions" between these domains. For example, it would be interesting to compare the conformations sampled by disordered regions in two MD trajectories, one started with K9 in the Rtt109 catalytic pocket and another started with K56 in the Rtt109 catalytic pocket.

2. The fit to SANS curves (Extended Fig 4b) is not great. This is, probably, because only the structured core of the complex was used in the fitting. A demonstration that inclusion of the disordered parts does not further improve the fitting, or some comments on this should be included in the manuscript.

3. In Methods, the authors state that they made resonance assignments for Vps75 (~260aa) and H3:H4 (> 200aa) proteins. The authors should specify how many and which resonances were assigned for each protein. Furthermore, the full 2D N15-HSQC and C13-HMQC spectra labeling the assigned peaks should be included in the manuscript.

4. The details of exactly which residues comprise each building block and what residues were considered flexible/rigid in the docking calculations are missing in the manuscript.

5. There is misprint Vps752 that marks Vps75 dimer.

Reviewer #2 (Remarks to the Author):

In this manuscript, the authors reported a study on the structure of the acetyltransferase Rtt109 in complex with Asf1 and Vps75 and the histone dimer H3:H4 using methyl-TROSY NMR, spin labeling and other biophysical techniques. They solved the solution-structure of the complex and investigated

the role of the disordered C-terminal domain of Vps75 in H3 K9-acetylation by Rtt109. The authors concluded that there are fuzzy electrostatic interactions between disordered domains of H3 and the Vps75 that help confine the H3 tail to a wide central cavity faced by the Rtt109 active site with minimal loss of entropy. They further speculate that such fuzzy interactions may represent a common mechanism of enzymatic reactions involving highly disordered substrates.

The study presented in this manuscript addressed an important question in the chromatin field regarding how specific post-translational modifications of histones are carried out by histone modification enzymes. It also shows an excellent example of the application of methyl-TROSY NMR method to the structural studies of large protein complexes. Overall, the work should have broad interests to people in the fields of chromatin and structural biology. The manuscript is well written. It is very suitable for publication in Nature Communications.

The claim that there are fuzzy interactions between disordered domains of H3 and Vps75 seems only weakly supported by the data (small chemical shift changes). The authors used a recently published study on the interactions between two intrinsically disordered molecules, linker histone H1 and its chaperone ProTa, to support their conclusion (ref. 39). However, a more recent study of the same complex has indicated that the earlier study overestimated the affinity by five orders of magnitude likely due to fluorescence dye labeling (Feng et al. Biochemistry, 2018; PMID:30430826). If the authors believe that the C-terminal tail of Vps75 has strong interactions with the N-terminal tail of H3, they should measure the binding affinity between the two fragments using NMR titration and/or ITC. Or they may want to tune down the claim.

The authors should state that they established a structural model of the complex rather than "solved the solution-structure" of the complex in the abstract. This is because they used the previously determined crystal structure of each component in their modeling instead of solving the structure of the whole complex de novo. It is still possible that the formation of the complex may lead to some conformation changes that may not be captured from the current study.

Answers to the reviewers' comments

Reviewer 1:

The authors performed MD simulations of the truncated complex Asf1–H3(35-135):H4–Rtt109–Vps75(1-225) that is missing both CTAD of Vps75 and NTD of H3 domains. These simulations reveal interaction between H3 and Vps75 that keeps K56 away from the Rtt109 catalytic pocket. However even more interesting would be simulation of the CTAD of Vps75 and NTD of H3 domains which could potentially shed some light on the "fuzzy interactions" between these domains. For example, it would be interesting to compare the conformations sampled by disordered regions in two MD trajectories, one started with K9 in the Rtt109 catalytic pocket and another started with K56 in the Rtt109 catalytic pocket.

While we agree that it would be interesting to derive a pool of meaningful transient conformations of the H3-NTD interacting with the Vps75-CTAD in the internal cavity of the complex, we believe that this is infeasible within a reasonable timeframe (2-6 months).

Classical all-atom MD simulations, as we performed for Extended Data Fig. 8), are not suitable to address this question. The simulations would need to be run for hundreds of microseconds to sample a representative — but still limited — subset of the conformational space; preliminary MD runs have led us to believe that our system would require even longer simulation times, as the disordered tails need to overcome energetic barriers to enter the internal cavity.

Faster methods to simulate this system include coarse-grained, replica-exchange or accelerated MD approaches. However, all these methods require accurate tuning of the initial parameters to avoid the introduction of biases. In addition, the calculations would need to be repeated for several starting conformations of the tails, where the tails are either inside or outside the central cavity.

Finally, once the simulations have been done, it would be necessary to validate them against experimental restraints. The only experimental restraints we have for this system are the ^{1}H and ^{15}N chemical shifts of the backbone amides, which are not straightforwardly applicable for validation of MD-derived conformers as they are highly dependent on many local structural parameters. Thus, in order to validate the MD simulations, we would need to acquire a completely new set of experimental parameters.

In conclusion, to undertake the request of the reviewer in a meaningful way, namely with validated MD simulations, requires an effort comparable to that of a new manuscript.

The fit to SANS curves (Extended Fig 4b) is not great. This is, probably, because only the structured core of the complex was used in the fitting. A demonstration that inclusion of the disordered parts does not further improve the fitting, or some comments on this should be included in the manuscript.

While the fits to the SANS curves have seemingly high χ^2 values (>1), we would argue that the fits are sufficiently good. First, the χ^2 values depend heavily on the exact estimation of the experimental errors. In SAS, it is known

that the values of the propagated errors depend on data reduction and can be either over- or under-estimated (Trewhella, J. *et al.* 2017 publication guidelines for structural modelling of small-angle scattering data from biomolecules in solution : an update. *Acta Cryst.* **D73,** 710–728 (2017)). Thus, it is recommended that χ^2 values be used to compare models with each other, rather than giving emphasis to their absolute values. As can be seen from the revised Extended Data Fig. 4b, all our datasets have very small statistical errors. All our samples were of sufficiently high concentrations to produce data with good signal-to-noise ratios; nevertheless, the curves show indications that the errors have been underestimated by the data reduction algorithms employed at the neutron facility where we recorded the data. For example, when we overlap the scattering points of the ^{2}H-Asf1–^{1}H-H3$^{35-135}$:H4–^{1}H-Rtt109–^{2}H-Vps75$_2^{1-225}$ curve, recorded in 42% D_2O, with the smoothed curve generated directly from the data, without fitting to any specific structural model, we see that the fluctuation of the intensities around the smoothed SANS curve is larger than the reported errors. This indicates that the errors of these points have been under-estimated by the data reduction algorithms employed at the neutron facility:

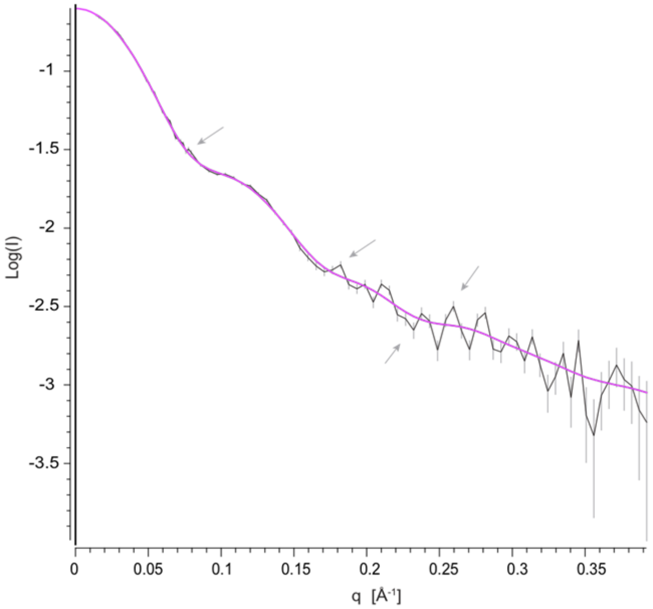

Despite the slightly elevated χ^2 values, the fitted curves match the experimental data and reproduce the correct positions of the characteristic minima and maxima. The curves have an excellent fit to the data at small angles, as demonstrated by the Guinier region shown in the insert of revised Extended Fig 4b. We would also like to stress that we report the χ^2 values, while all χ, except for one, have values in the 1.4 – 2.5 range.

The ^{1}H-Asf1–H3$^{35-135}$:H4–^{1}Rtt109–Vps75$_2^{1-225}$ curve (grey in Extended Data Fig. 4b) is the only curve that does not represent a good fit by visual inspection. The suboptimal fit in the q range of 0.08 – 0.15 is caused by the ‘smearing’ effect due to a limited wavelength resolution (Δλ/λ) of 10%. This phenomenon is not observed for X-rays (i.e. SAXS) at synchrotrons or home sources, where a single, well-defined wavelength is used. The SANS curves

with pronounced maxima and minima (such as the curve in question) are the most sensitive to this effect. If one takes the wavelength resolution into account, the fit improves substantially (see below). In the revised version of Extended Data Fig. 4b, the ^{1}H-Asf1–H3$^{35\text{-}135:}$H4–^{1}Rtt109–Vps75$_2$$^{1\text{-}225}$ curve is now fitted taking the wavelength resolution into account.

Addition of the missing flexible parts, such as H3$^{35\text{-}60}$ and Rtt109$^{418\text{-}436}$ further improves the fit. However, different conformations of H3$^{35\text{-}60}$ would have different effects on the curve. Due to the small influence of these disordered regions on the curve, in comparison to the “smearing” effect, we refrain from commenting thereon in the revised version of the manuscript, but include the fits to the SANS data that take the wavelength resolution into account.

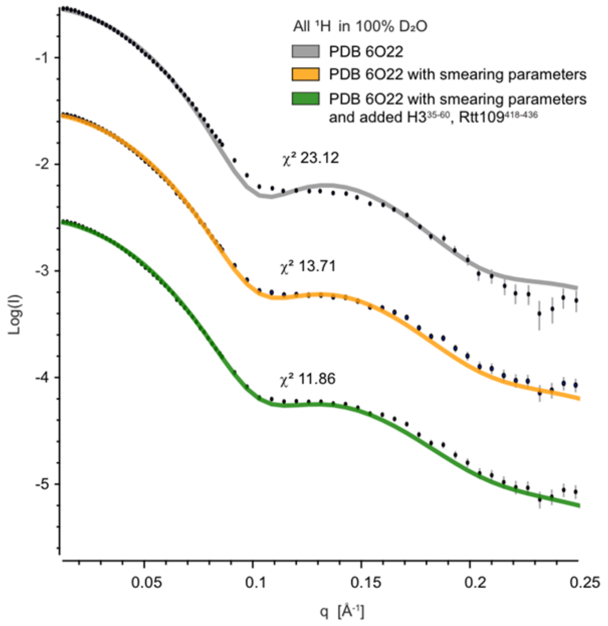

In Methods, the authors state that they made resonance assignments for Vps75 (~260aa) and H3:H4 (> 200aa) proteins. The authors should specify how many and which resonances were assigned for each protein.

This information is now provided in the Online Methods and marked in red.

Furthermore, the full 2D N15-HSQC and C13-HMQC spectra labeling the assigned peaks should be included in the manuscript.

These spectra are now provided as Extended Data Figs. 10 and 11.

The details of exactly which residues comprise each building block and what residues were considered flexible/rigid in the docking calculations are missing in the manuscript.

This information has been added to Online Methods and marked in red.

There is misprint Vps75$_2$ that marks Vps75 dimer.

The wording “Vps75$_2$ dimer” has been changed to “Vps75 dimer”.

Reviewer 2

The claim that there are fuzzy interactions between disordered domains of H3 and Vps75 seems only weakly supported by the data (small chemical shift changes). The authors used a recently published study on the interactions between two intrinsically disordered molecules, linker histone H1 and its chaperone ProTa, to support their conclusion (ref. 39). However, a more recent study of the same complex has indicated that the earlier study overestimated the affinity by five orders of magnitude likely due to fluorescence dye labeling (Feng et al. Biochemistry, 2018; PMID:30430826). If the authors believe that the C-terminal tail of Vps75 has strong interactions with the N-terminal tail of H3, they should measure the binding affinity between the two fragments using NMR titration and/or ITC. Or they may want to tune down the claim.

The values of the CSPs are not indicative of the strength of the interaction, but rather of the differences in the chemical environment. In the case of fuzzy interactions, we expect small chemical shift changes as the atomic interactions are different in each of the conformations comprising the ensemble, causing different CSPs. Thus, we observe an average of the CSPs produced by each conformer of the "fuzzy" complex.

In any case, we did not intend to make any statement regarding the strength of the fuzzy interaction in our complex, as there is no straightforward way by which it can quantified. The strength of the interaction between the two disordered tails measured by calorimetry would not be representative of the strength of the interaction in the cavity, where the Vps75-CTAD also interacts with the histone DNA-binding surface and may thereby adopt a preferred set of transient folds. NMR is also not useful to measure the strength of the fuzzy interaction as the complex assembles tightly in a 1:1:1:2 stoichiometry, independently of the presence of the tails.

We apologize if we have given the impression that we define our interaction as "strong". We have carefully re-read the discussion and are not of the opinion that there are any inferences regarding the strength of the fuzzy interactions. To eliminate any confusion however, and in light of the recent paper cited by the reviewer, which we now also cite, we avoid the term "high-affinity interaction" when we refer to the complex described by Borgia *et al.*

The authors should state that they established a structural model of the complex rather than "solved the solution-structure" of the complex in the abstract. This is because they used the previously determined crystal structure of each component in their modeling instead of solving the structure of the whole complex de novo. It is still possible that the formation of the complex may lead to some conformation changes that may not be captured from the current study.

This has been reformulated throughout the manuscript, as suggested by the reviewer.

REVIEWERS' COMMENTS:

Reviewer #1 (Remarks to the Author):

The authors have addressed my concerns. I appreciated that new MD studies may be beyond the scope of this study.

Reviewer #2 (Remarks to the Author):

I am satisfied with the revision.
